# Ionotropic Chemosensory Receptors Mediate the Taste and Smell of Polyamines

**DOI:** 10.1371/journal.pbio.1002454

**Published:** 2016-05-04

**Authors:** Ashiq Hussain, Mo Zhang, Habibe K. Üçpunar, Thomas Svensson, Elsa Quillery, Nicolas Gompel, Rickard Ignell, Ilona C. Grunwald Kadow

**Affiliations:** 1 Sensory Neurogenetics Research Group, Max-Planck Institute of Neurobiology, Munich, Germany; 2 Unit of Chemical Ecology, Department of Plant Protection Biology, Swedish University of Agricultural Sciences, Alnarp, Sweden; 3 Faculty of Biology, Ludwig-Maximilians-University Munich, Munich, Germany; Vlaams Instituut voor Biotechnologie and Katholieke Universiteit Leuven, BELGIUM

## Abstract

The ability to find and consume nutrient-rich diets for successful reproduction and survival is fundamental to animal life. Among the nutrients important for all animals are polyamines, a class of pungent smelling compounds required in numerous cellular and organismic processes. Polyamine deficiency or excess has detrimental effects on health, cognitive function, reproduction, and lifespan. Here, we show that a diet high in polyamine is beneficial and increases reproductive success of flies, and we unravel the sensory mechanisms that attract *Drosophila* to polyamine-rich food and egg-laying substrates. Using a combination of behavioral genetics and in vivo calcium imaging, we demonstrate that *Drosophila* uses multisensory detection to find and evaluate polyamines present in overripe and fermenting fruit, their favored feeding and egg-laying substrate. In the olfactory system, two coexpressed ionotropic receptors (IRs), IR76b and IR41a, mediate the long-range attraction to the odor. In the gustatory system, multimodal taste sensation by IR76b receptor and GR66a bitter receptor neurons is used to evaluate quality and valence of the polyamine providing a mechanism for the fly’s high attraction to polyamine-rich and sweet decaying fruit. Given their universal and highly conserved biological roles, we propose that the ability to evaluate food for polyamine content may impact health and reproductive success also of other animals including humans.

## Abstract 2

Polyamines are beneficial nutrients for reproduction, cognition, and lifespan. This study reveals that insects are highly attracted to them and identify their sources through an ancient class of ionotropic receptors on their smell and taste organs.

## Author Summary

Animals, including humans, evaluate food by its smell and taste. Odors and tastes not only signal the presence of food, they also reveal details about the type and amount of nutrients contained in it. A preference for certain foods frequently reflects the specific metabolic needs of an animal. Among the important but less known compounds that animals consume with their diet are polyamines. These pungent smelling molecules are essential for reproduction, development, and cognition. Interestingly, they are also produced by the cell and body, but their levels decline with age. A diet high in polyamines can improve age-related memory deficits and loss of fertility. We have used the model fly *Drosophila melanogaster* to unravel if and how animals detect polyamines in their food and environment, and which role this detection plays in their food choice behavior. Polyamine levels are particularly high in the fly’s favorite food and egg-laying substrate, overripe and decaying fruit. We found that food supplemented with polyamines indeed improves the reproductive success of a fly couple. We show that *Drosophila* is highly attracted to polyamines and uses them to identify promising egg-laying and feeding sites. It detects them through an ancient clade of receptor proteins on its olfactory and taste organs. We speculate that other animals can also detect polyamines and use their smell and taste to identify sources of these beneficial nutrients.

## Introduction

Animals make use of all of their senses while searching and evaluating food. For most, smell and taste are the major modalities to assess the quality and nutritional value of food. While odors help the animal to track down food over long distances, short-range evaluation using the sense of taste is ultimately crucial for the decision whether to feed or to lay an egg [1,2]. In general, animals prefer calorie-dense and sweet foods to bitter foods (see for instance [3]). In addition, animals need to consume food containing other important nutrients. Among the vital dietary constituents are polyamines [4]. Polyamines, most notably putrescine, spermine, and spermidine, are essential for basic cellular processes such as cell growth and proliferation, and are of specific importance during reproduction [5,6]. While polyamines can be generated by endogenous biosynthesis or microbes in the gut, a significant fraction comes from the diet [4]. Animal products, soybeans, or certain fruits are rich sources of polyamines [4]. In cells, these polycations are bound to nucleic acids, proteins, and phospholipids, where they participate in fundamental cellular processes such as DNA replication, RNA translation, and mitosis [6,7]. Polyamine deficiency can have fatal consequences on reproductive success [5]; and low polyamine levels have been linked to neurodegenerative diseases and ageing [8]. Yet, high polyamine concentrations are found in cancer cells suggesting that their excess could be unhealthy [6]. Notably, the enzymes that generate endogenous polyamines decline with ageing [5–7]. And the exogenous supply through high polyamine diets can have beneficial effects on ageing, memory loss, and reproduction in a variety of model species and humans [4,8–11]. Given their beneficial but also detrimental roles, food industry has consequently measured the amount of polyamines in many food items [12]. Whether animals can directly evaluate polyamine content for instance through their taste organ is not known. The smell of polyamines, however, can be detected by animals, including humans; it is strongly pungent and at higher concentrations unpleasant to humans. There is circumstantial evidence indicating that insects may detect the smell and taste of polyamines. For instance, *Calliphora* blowflies are attracted to decaying flesh and lay their eggs exclusively into corpses. This attraction could be due to the carrion smell of cadaverine [13]. *Drosophila* flies are notorious for their attraction to overripe and fermenting fruit [14,15]. The amount of polyamines increases dramatically in fruit within days after harvest [16] and during fermentation [17]. Therefore, for these flies, polyamines are candidate molecules for the detection of beneficial feeding and egg-laying sites. This may also hold true for females of other insects. For instance, females of the dengue fever vector, the mosquito *Aedes aegypti* typically lay their eggs in batches in standing waters such as flowerpot plates with decaying organic and polyamine-rich materials [18,19]. Although an olfactory receptor, trace amine-associated receptor 13c (TAAR13c), for one polyamine, cadaverine, was recently described in zebrafish [20], no taste receptor has been identified so far. Chemosensory receptors for the detection of polyamines remain uncharacterized in insects.

To detect chemosensory stimuli animals use highly specialized families of receptor proteins that are present in sensory neurons on peripheral taste or smell organs [21]. Given their importance in animal life, a large effort goes into identification of these receptors and their putative odors or tastes. *D*. *melanogaster* has proven to be a useful model in matching olfactory and gustatory receptors (GRs) to their ligands and has contributed much to our understanding of chemosensory coding in the nervous system [22].

Insects possess three classes of olfactory receptors (ORs): the classical ORs, the more recently described but evolutionarily older family of ionotropic receptors (IRs), and a few GRs [23–26]. Each olfactory sensory neuron (OSN) is located in a sensillum on either antenna or maxillary palp and expresses a specific type or very small combination of receptors, which are tuned to a narrow group of molecules. All OSNs that express the same receptor project their axons to one of ~50 glomeruli in the antennal lobe (AL) in the central brain [27]. This highly conserved architecture allows the translation of a nonspatial sensory cue into a highly organized spatial map and provides the logic for odor coding [21]. Upon additional local processing at the level of the AL, the odor information is sent via projection neurons (PNs) to two main higher brain centers, the mushroom body and the lateral horn [28]. While many of the ~45 ORs have been deorphanized, ligands for a number of IRs remain uncharacterized [22,29]. Previous work showed that most of the IR OSNs express one of the putative coreceptors IR8a or IR25a [30,31]. Among the deorphanized IRs is IR92a, the receptor for ammonia and small amines [32]. The behavioral role of most IRs, however, remains elusive with few exceptions such as IR84a and IR64a [29,33,34].

Gustatory receptor neurons (GRNs), in contrast to OSNs, are found on many peripheral as well as internal organs [35]. On the external sensory organs, GRN-containing sensilla are mainly found on the labellum, the legs, and the wing margins [35]. The labellum carries ~60 morphologically distinct sensilla with four GRNs each that are tuned to distinct flavors such as sweet, salty, water (appetitive) or bitter, and acidic (noxious). While GRs form the best-characterized family of taste receptors to date [22,36], more recent members of the IRs have been implicated in the sensation of tastants [37–39]. For instance, IR76b was shown to be essential for the detection of appetitive concentrations of salt [40]. Interestingly, IR76b is also expressed in GRNs that do not detect salt, but a role for these neurons has not been assigned yet. Finally, increasing evidence suggests that dietary amines can be tasted, but receptors have not been identified yet [37]. Peripheral GRNs project to the central brain or in the case of GRNs on tarsae or wings, also to the ventral nerve cord [35]. In the central brain, the distinct patterns of innervation in an area called the subesophageal zone (SEZ) by bitter and sweet neurons indicated a taste map similar to but less structured than the map found in the AL of the olfactory system [41]. In contrast to the olfactory system, however, higher order processing of tastes is still not well understood [42].

We have analyzed at the sensory, molecular, and behavioral levels how polyamines guide insect preference behavior. First, we demonstrate that a polyamine-rich diet significantly increases the number of progeny of flies. Second, we show that flies can find and evaluate polyamine-rich feeding and egg-laying sites using their senses of smell and taste. We characterize the polyamine receptors and demonstrate an essential role for specific IRs in olfactory and gustatory organs. Altogether, our data characterize sensory receptors for polyamines and their behavioral role in insects and indicate that the ability to sense polyamines promotes reproductive success and survival.

## Results

### Polyamine-Rich Diet Increases Reproductive Success of *Drosophila*

**Fig 1:**
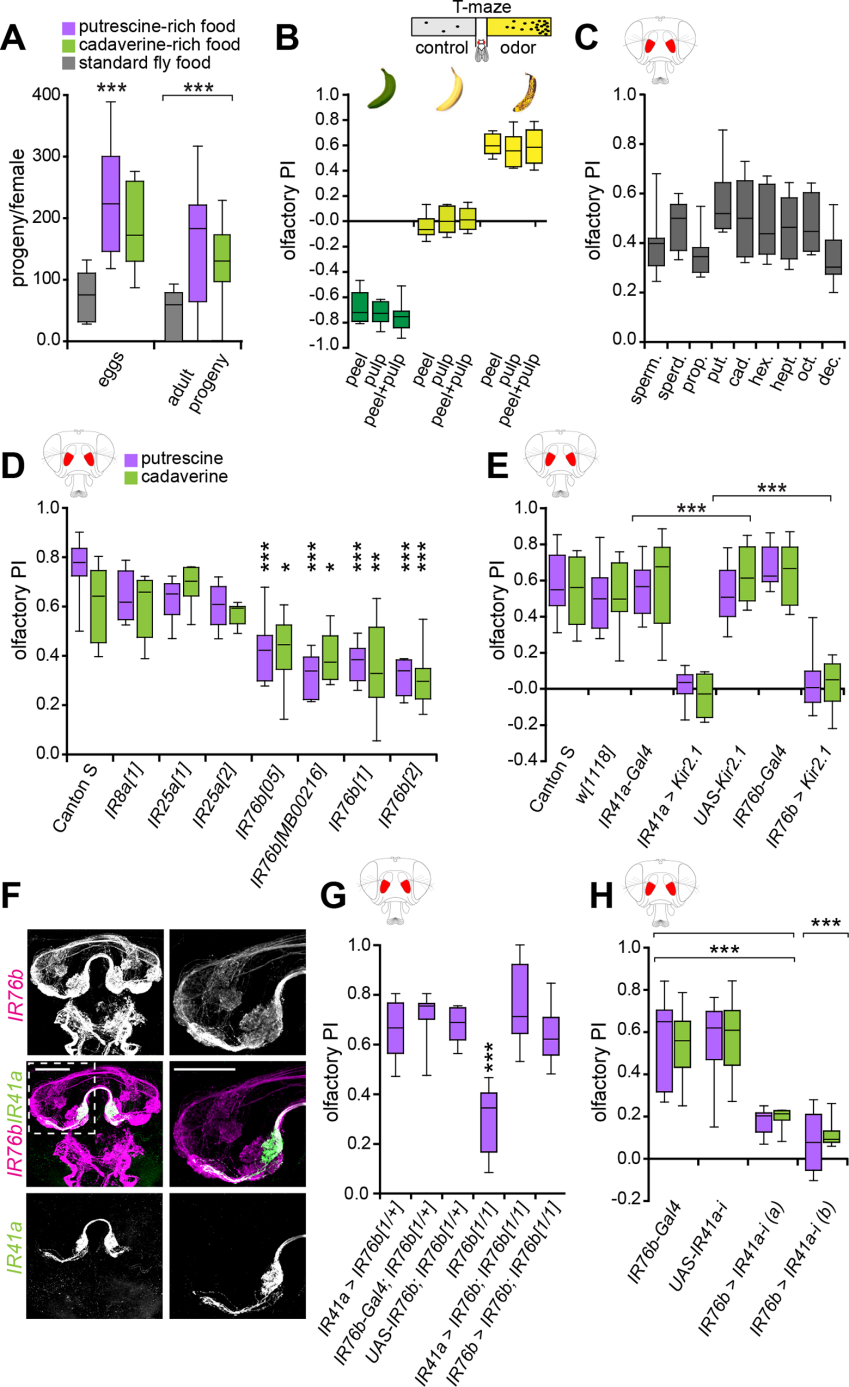
IR41a and IR76b mediate olfactory attraction to polyamines. (A) A diet high in polyamines increases reproductive success. Single males were crossed with single females in two different conditions for 4 d. Standard fly food and fly food with additional putrescine or cadaverine solution (~2.5 mmol polyamine/l of food). The number of eggs laid per single female and the number of eclosed flies per single female was quantified. Box plot show median and upper/lower quartiles (*n* = 8, 2 flies/trial 1 ♀ and 1 ♂). (B) Schematic illustration of the T-maze assay (top). *Drosophila* is attracted to the smell of overripe banana. Bars show olfactory preference index (PI) of wild type Canton S flies to peel, pulp, and peel + pulp of green banana, just yellow banana and brown-speckled banana, respectively. *y*-axis value of 0 indicates indifference while positive values indicate the degree of attraction and negative values indicate aversion. Box plots show median and upper/lower quartiles (*n* = 8, 60 flies/trial, 30 ♀ and 30 ♂). (C) Flies are attracted to several polyamines (of chain lengths C3-C10) with different sensitivities. Graph shows olfactory PI of wild type flies to 1 mM spermine (sperm.), spermidine (sperd.), diaminopropane (prop.), putrescine (put.), cadaverine (cad.), diaminohexane (hex.), diaminoheptane (hept.), diaminooctane (oct.), and diaminodecane (dec.), respectively. Box plots show median and upper/lower quartiles (*n* = 8, 60 flies/trial, 30 ♀ and 30 ♂). (D) Olfactory PI of Canton S (control) and IR mutant flies to putrescine and cadaverine in the T-maze assay. Box plots show median and upper/lower quartiles (*n* = 8, 60 flies/trial, 30 ♀ and 30 ♂). Asterisks denote significant reduction in olfactory preference to putrescine and cadaverine. (E) Bar graph shows olfactory PI of *IR41a-Gal4;UAS-Kir2*.*1* and *IR76b-Gal4;UAS-Kir2*.*1* with Canton S and genetic controls to putrescine and cadaverine in the T-maze assay. Asterisks denote a significant reduction in olfactory preference to putrescine and cadaverine. Box plots show median and upper/lower quartiles (*n* = 8, 60 flies/trial, 30 ♀ and 30 ♂). (F) Costaining of *IR41a-Gal4;UAS-mCD8GFP* (green) and *IR76b-QF;QUAS-mdtomato* (magenta) in the fly brain. The boxed region is centered on the AL, which is shown enlarged in the right panels. IR41a and IR76b expressing axons coinnervate a single glomerulus (VC5) in the AL. (G) IR76b is necessary in IR41a neurons to mediate the behavioral response to polyamine odor. IR76b was re-expressed in the *IR76b* mutant background using *IR76b-Gal4* or *IR41a-Gal4*. While *IR76b* mutants show a significantly reduced response to putrescine, re-expression of IR76b in either IR76b or IR41a neurons fully rescued this defect. (H) IR41a receptor is essential for polyamine attraction. Bar graphs show olfactory PIs of flies carrying *IR76b-Gal4;UAS-IR41a-RNAi* of two different RNAi transgenes flies and their genetic controls to putrescine and cadaverine in the T-maze assay. Box plots show median and upper/lower quartiles (*n* = 8, 60 flies/trial, 30 ♀ and 30 ♂). All *p*-values were calculated via two-way ANOVA with the Bonferroni multiple comparison posthoc test (ns > 0.05, *p ≤ 0.05, **p ≤ 0.01, ***p ≤ 0.001). In all figures, asterisks above a single bar refer to *p*-values of comparison to the control (first bar of the panel). Lines joining two bars or groups of bars denote all other comparisons.

Given the evidence that polyamines are vital molecules during reproduction, we asked whether males and females feeding on polyamine-rich food would produce more offspring compared to flies on standard fly food (see Materials and Methods). Therefore, we crossed single males to single females in two different conditions—on standard fly food and fly food that had been supplemented with putrescine or cadaverine solution (~2.5 mmol polyamine/l of food). After 4 d, the parental generation was discarded, and the number of eggs laid was quantified. In addition, once these eggs had developed into flies, these were counted again. Females on polyamine-enriched food laid ~3 times the amount of eggs compared to females on standard food. Similarly, fly pairs fed a high-polyamine diet had ~3 times more offspring than the couples on standard fly food (Fig 1A). Thus, it indeed appeared that polyamine-enriched food was beneficial for reproduction of flies similarly as what has been suggested for other species such as humans.

### *Drosophila* Is Strongly Attracted to Volatile Polyamines

*D*. *melanogaster* flies are notorious for being attracted to, and for laying their eggs into, decaying fruits [14]. We asked whether this preference was rooted in the need to consume polyamines, present in fermenting fruit. First, we quantified the attraction of male and female flies to fruits at different stages of maturity with a laboratory choice assay, the T-maze. We found that flies show a strong aversion to green bananas, are indifferent to yellow bananas, but are highly attracted to the same batch of overripe bananas 5–7 d later (Fig 1B) [43]. Next, we tested whether *Drosophila*’s attraction to decaying fruit could, in part, be attributed to increased concentrations of polyamines produced during ripening and decay [16]. Running the same T-maze assays with different polyamines, we found that female and male flies were strongly attracted to the odor of spermine (sperm.), spermidine (sperd.), diaminopropane (prop.), putrescine (put.), cadaverine (cad.), diaminohexane (hex.), diaminoheptane (hept.), diaminooctane (oct.), and diaminodecane (dec.) (Fig 1C). The responses were dose-dependent with 1 mM (~10 ppm) eliciting the strongest attraction compared to lower as well as higher concentrations (S1A Fig; 1 μM–1 M). This concentration roughly corresponded to the amount of putrescine found in fermented banana (~0.9 mmol/kg) or fresh oranges (~1.3 mmol/kg) [12]. We observed the same attraction when single female flies were assayed in the T-maze, suggesting that individual flies perceive and are attracted to the odor (S1B Fig).

To uncover the neural basis of the fly’s attraction to this class of important nutrients, we sought to identify the receptor for polyamine sensation. Preference to a chemical in the T-maze is typically mediated by the olfactory system. Indeed, flies with surgically removed antennae, the main olfactory organ, lost their T-maze preference for polyamines (S1C Fig). Previous reports indicated that OR- and IR-expressing OSNs respond to putrescine in single sensillum recordings (SSR) [23,44–46]. From these two receptor classes, the entire OR system can be impaired at once by mutating the obligatory OR coreceptor (Orco) [47]. *Orco* mutant flies maintained normal attraction to putrescine (S1D Fig), excluding this family of receptors from our search as suggested before [46]. We then analyzed the requirement of IRs. To suppress all IR-mediated chemosensation, we relied on *atonal* (*ato*) mutants, as they fail to develop IR-expressing coeloconic sensilla [23,48,49]. *ato* mutant flies did not show any preference for putrescine or cadaverine in the T-maze (S1E Fig). We concluded that IRs mediate attraction to volatile polyamines. To identify specific IRs, we carried out a small genetic screen using loss of function of single IRs. From all IR mutants tested, including the two putative coreceptors *IR8a* and *IR25a*, only *IR76b* mutants showed a significant reduction in polyamine attraction in the T-maze (Fig 1D, S1F Fig). To confirm a requirement for IR76b, we silenced the activity of IR76b neurons by expressing the inward-rectifier potassium channel Kir2.1 [50]. Flies of the genotype *IR76b-Gal4;UAS-Kir2*.*1* showed a strong impairment in attraction to putrescine or cadaverine (Fig 1E).

It has been suggested that IRs may form functional heteromers in olfactory neurons, similarly to ORs [30]. Furthermore, IR76b as judged by reporter expression using a previously characterized *IR76b-Gal4* transgene [40] was expressed in multiple types of OSNs with axons innervating four glomeruli strongly and three weakly in the AL (Fig 1F; [46]). This strengthened the notion that another receptor might be used in conjunction with IR76b as previously hypothesized [46]. Among the OSNs previously shown to respond to putrescine [46] is a subset of IR41a-expressing neurons, housed in ac2 sensilla [31,46]. We blocked the activity of IR41a neurons with Kir2.1 *(IR41a-Gal4;UAS-Kir2*.*1)* and found that similar to IR76b neuron silencing, these flies showed no attraction to polyamines (Fig 1E). Using double labeling with *IR41a-Gal4* and *IR76b-QF*, which labels the same neurons as *IR76b-Gal4* (S2 Fig and see also Silbering et al. [46]), we found that a small subset of OSNs innervating a ventral and central (VC5) glomerulus coexpresses IR41a and IR76b (Fig 1F).

To obtain more direct evidence of a requirement of IR76b in IR41a neurons, we re-expressed IR76b in the *IR76b* mutant background selectively in IR41a or in all IR76b neurons (Fig 1G). As expected, re-expression of IR76b in IR76b neurons fully rescued the flies’ attraction to polyamine odor (*IR76b-Gal4;UAS-IR76b;IR76b*^*1*^, Fig 1G). Importantly, the same rescue was observed when we re-expressed IR76b selectively in IR41a neurons (*IR41a-Gal4;UAS-IR76b;IR76b*^*1*^, Fig 1G). In a reciprocal experiment to test the role of IR41a in these neurons, we used RNAi to knockdown IR41a in IR76b-expressing neurons (*IR76b-Gal4;UAS-IR41a-i*) and assayed the effect in the T-maze. Knockdown of IR41a using two different RNAi transgenes reduced attraction to polyamines significantly as compared to control flies (Fig 1H).

These data, taken together, provide strong evidence that IR41a/IR76b coexpressing neurons are necessary and sufficient to mediate polyamine attraction.

### IR76b Is Required for the Polyamine Odor Response of IR41a OSNs

**Fig 2:**
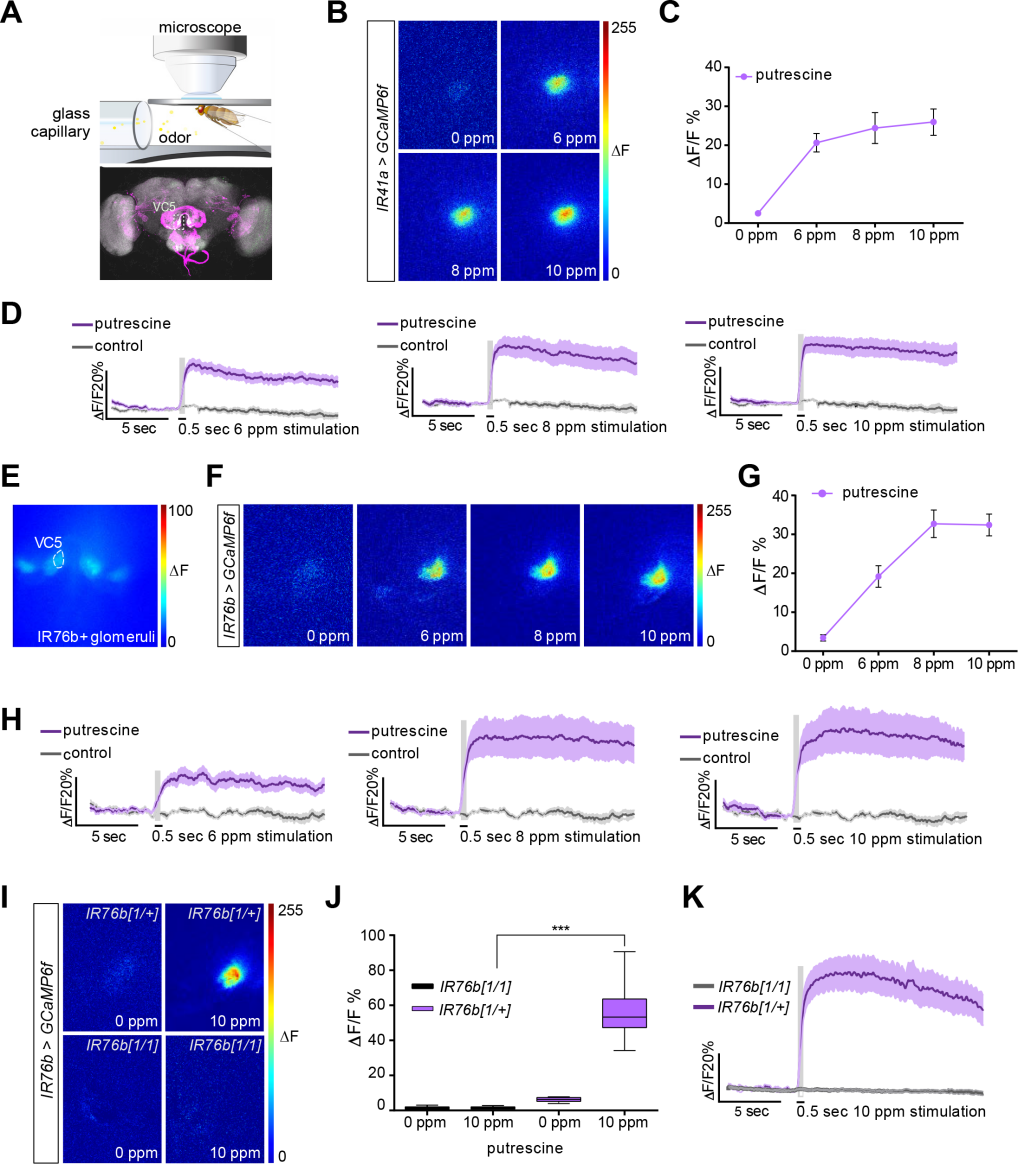
IR76b is required for the polyamine odor response of IR41a OSNs. (A) In vivo imaging setup illustration (top). Illustrative confocal image showing the IR41a and IR76b OSN innervating glomeruli pattern (bottom). VC5 is the glomerulus innervated by the polyamine-responding IR41a sensory neurons. (B–D) In vivo calcium imaging of *IR41-Gal4;UAS-GCaMP6f* flies stimulated with water and 6, 8, and 10 ppm putrescine, respectively. Please note that that based on PID measurements, 10 ppm most closely represented the 1 mM concentration used in behavioral experiments due to the technical differences of odor application. (B) Representative pseudocolor images showing the response to water and increasing doses of putrescine, respectively. (C) Quantification of peak ΔF responses of the VC5 glomerulus (*n* = 9). (D) Average activity trace of VC5 glomerulus upon polyamine stimulation in %ΔF/F. (E) Prestimulation fluorescence micrograph showing IR76b positive glomeruli. (F–H) In vivo calcium imaging of *IR76b-Gal4;UAS-GCaMP6f* flies stimulated with water and 6, 8, and 10 ppm putrescine, respectively. (F) Representative pseudocolor images showing the response to increasing doses of putrescine. (G) Quantification of peak ΔF responses of the VC5 glomerulus (*n* = 6). (H) Average activity trace of VC5 glomerulus in %ΔF/F. (I–K) In vivo calcium imaging of *IR76b-Gal4*,*UAS-GCaMP6f;IR76b*^*1*^ and heterozygous control flies stimulated with water and 10 ppm putrescine, respectively. (I) Representative pseudocolor images showing the response in the VC5 glomerulus. (J) Quantification of peak ΔF responses in mutant and control flies. Boxes show median and upper/lower quartiles, and whiskers show minimum/maximum values. ***p < 0.001 by unpaired *t* test with Welch correction (*n* = 6). (K) Average activity trace of VC5 glomerulus. (D, H, K) The gray column represents the 0.5 second stimulation period. Dark colored line is the average response and the light shade is the standard error of the mean (SEM).

To strengthen the evidence for a role of IR41a/IR76b OSNs in the detection of polyamine odor, we used in vivo calcium imaging as a proxy of neuronal activity of these OSNs. To this end, we expressed the genetically encoded calcium indicator GCaMP6f [51] under the control of *IR76b-Gal4* or *IR41a-Gal4* (*IR-Gal4;UAS-GCaMP6f*) and recorded increases in intracellular calcium levels in response to the polyamine odor stimulus at the level of the axon terminals in the AL in the brain (Fig 2A). When GCaMP was expressed exclusively in IR41a neurons, the innervated glomerulus strongly responded to putrescine in a concentration-dependent manner (Fig 2B–2D; see also [46]). Similarly, only a single glomerulus, the one innervated by IR41a/IR76b neurons, responded strongly to polyamine odor when GCaMP was expressed in all IR76b neurons (Fig 2E–2H). An independent IR76b, but not IR41a, neuron-innervated glomerulus, by contrast, did not respond significantly to polyamines (S3A–S3C Fig). Notably, the response was very long lasting (> 15 s, Fig 2D and 2H). Photoionization detector (PID) measurements suggested that odor might have been released into the airstream for up to 4 s with a 500 ms stimulus, because polyamine leaves a trace in the delivery line, which is cleaned out by air. This could, in part, explain this long-lasting response. Alternatively, this prolonged response could be a feature of some IR neurons as has been observed for other odors [32].

To test a requirement for IR76b in the observed odor response, we analyzed *IR76b*^*1*^ mutant flies. The response of the IR41a glomerulus was strongly reduced in *IR76b* loss of function mutants (*IR76b-Gal4;UAS-GCaMP6f;IR76b*^*1*^) confirming the essential role of IR76b for polyamine odor detection (Fig 2I–2K).

These experiments suggest that IR41a and IR76b function in the same neurons as polyamine receptors used by flies to detect polyamine-rich food sources such as overripe fruit.

### Taste Neurons Mediate Short-Range Egg-Laying Choices on Polyamines

**Fig 3:**
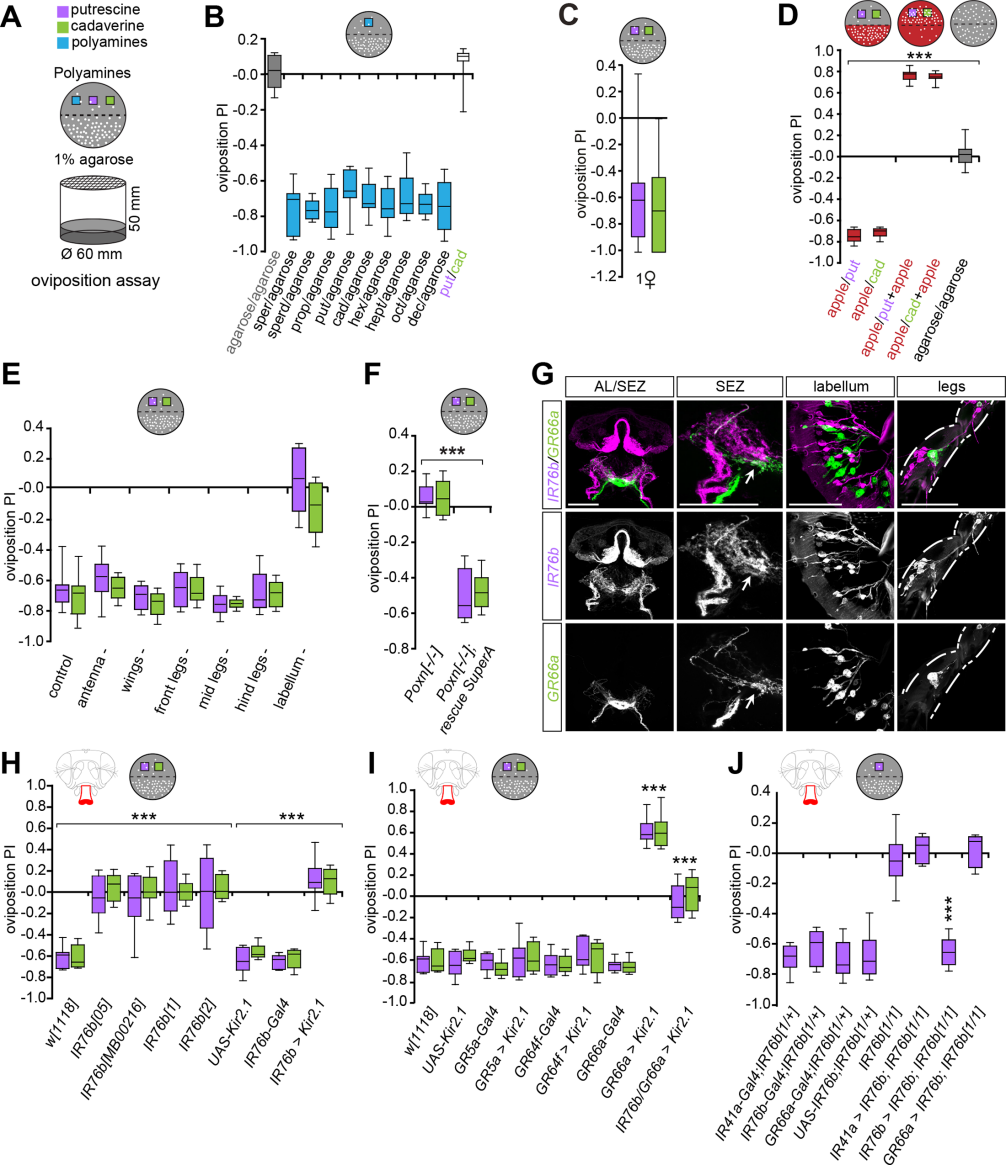
Oviposition site choice requires IR76b and bitter taste neurons. (A) Schematic drawing of the oviposition assay setup (bottom) and a sample plate used to calculate the oviposition preference (top). *D*. *melanogaster* evaluates polyamine levels during egg-laying choices. The egg-laying plate halves contain 1% low melting agarose alone or agarose supplemented with a specific polyamine (purple/green boxes) in all cases with the exception of Fig 3D. (B–J) Box plots show oviposition PI of flies. *y*-axis value of 0 indicates indifference, while positive values indicate the degree of attraction and negative values indicate aversion. (B) Oviposition assay using plain agarose versus agarose + different polyamines at 1 mM. Box plots show median and upper/lower quartiles (*n* = 8, 60 ♀/trial). (C) Same assay as in B with single females showing group-independent decision-making to polyamines. (*n* = 30, 1 ♀ flies/trial). (D) Polyamines increase attractiveness of fruit. Oviposition PI of females for putrescine or cadaverine (grey plate) versus apple juice (red plate). While apple juice is more attractive than plain putrescine or cadaverine, apple juice supplemented with polyamine is more attractive than apple juice alone. Box plots show median and upper/lower quartiles (*n* = 8, 60 ♀/trial). (E) Oviposition assay (agarose versus putrescine or cadaverine) with females missing either antennae, wings, different tarsae (legs), or labellum compared to intact flies (control). Flies missing the labellum show no preference, while all other ablations had no effect on the PI. Box plots show median and upper/lower quartiles (*n* = 8, 60 ♀/trial). (F) Oviposition PI of loss of function *Poxn* females (*Poxn*^*-/-*^) and *Poxn* rescue construct (SuperA-158 (53)) for putrescine and cadaverine. Box plots show median and upper/lower quartiles (*n* = 8, 60 ♀/trial). (G) Expression of GR66a (*GR66a-Gal4;UAS-mCD8GFP*, green) and IR76b (*IR76b-QF;QUAS-mtdTomato-3xHA*, magenta) in the AL, SEZ, labellum and legs. GR66a and IR76b are not expressed in the same taste neurons but innervate neighboring areas in the SEZ (arrow). (H) *IR76*b mutants lose their preference behavior to polyamine taste (*IR76b*^*05*^, *IR76b*^*MB00216*^, *IR76b*^*1*^, *IR76b*^*2*^), *IR76b-Gal4;UAS-Kir2*.*1*, and appropriate genetic controls. Box plots show median and upper/lower quartiles (*n* = 8, 60 ♀/trial). (I) Two taste receptors mediate oviposition preference. Oviposition PI of silenced sweet tasting GRs (*GR5a-Gal4;UAS-Kir2*.*1* and *GR64f-Gal4;UAS-Kir2*.*1*), and inactivated bitter tasting receptor neurons (*GR66a-Gal4;UAS-Kir2*.*1*) and appropriate controls. Silencing of bitter neurons makes polyamines attractive, while silencing sweet neurons has no effect. This attractiveness is dependent on the activity of IR76b neurons as *GR66a-Gal4*,*IR76b-Gal4;UAS-Kir2*.*1* flies show no preference behavior. Box plots show median and upper/lower quartiles (*n* = 8, 60 ♀/trial). (J) IR76b is required to mediate the behavioral response to polyamine odor. IR76b was re-expressed in the *IR76b* mutant background using *IR76b-Gal4*, *IR41a-Gal4 or GR66a-Gal4*. While *IR76b* mutants show no preference behavior to putrescine, re-expression of IR76b in IR76b but not in IR41a or GR66a neurons fully rescued this defect. Box plots show median and upper/lower quartiles (*n* = 8, 60 ♀/trial). All *p*-values were calculated via two-way ANOVA with the Bonferroni multiple comparison posthoc test (ns > 0.05, *p ≤ 0.05, **p ≤ 0.01, ***p ≤ 0.001). In all figures, asterisks above bars refer to *p*-values of comparison to wild type control (first bar of the panel). Lines joining two bars or groups of bars denote all other comparisons.

Our data indicated that flies are attracted to a source of volatile polyamine such as overripe fruit through specific olfactory neurons. Furthermore, we showed that females on polyamine-enriched food laid more eggs and had more offspring than females on standard food (see Fig 1A). We therefore asked if females use polyamines as a hallmark of beneficial egg-laying sites. To analyze this, we quantified the number of eggs laid on a polyamine-rich but otherwise plain, sugar-free substrate (polyamine and 1% agarose) versus a control substrate (1% agarose) in an oviposition assay (Fig 3A and 3B). Surprisingly, and in contrast to their olfactory preference, female flies avoided the polyamine-rich substrate and laid the majority of eggs on the polyamine-free site (Fig 3B, S4A–S4C Fig). Single females made the same choice as groups of females and laid their eggs away from polyamines showing that this aversion was not a consequence of overcrowding (Fig 3C, S4D Fig). While it was previously suggested that female flies avoid laying their eggs directly into feeding substrates [52], the apparent dislike of the beneficial polyamine-rich substrates as oviposition sites was surprising. This behavior could reflect the rather artificial assay conditions, where flies chose between polyamine-rich and polyamine-free but an otherwise taste- or odorless substrate. Because in a decaying fruit, polyamines appear together with other food odors and tastes, we reasoned that females might find them more appealing for oviposition when combined to other chemosensory cues. We tested this by mixing either of two polyamines, putrescine or cadaverine, with apple juice and gave the flies the choice to lay their eggs either on apple juice alone or on the mixture. While flies prefer to lay their eggs on apple juice compared to pure polyamine, they strongly preferred the mixture of apple juice and polyamines to apple juice alone (Fig 3D, S4E Fig). Similarly, flies laid significantly more eggs onto a substrate that contained sugar and putrescine than on sugar alone (S4F Fig). Thus, polyamines are not only beneficial for egg-laying and increase the number of progeny, they also provide and enhance appealing landmarks for oviposition.

The data highlighted that females use combinatorial sensory cues to decide where to lay an egg. We therefore examined which sensory modalities contributed to this short-range decision during oviposition. To facilitate the dissection of sensory mechanisms, we returned to assays using polyamine alone (agarose + polyamine versus agarose) and first determined the role of different sensory organs in polyamine choice during egg-laying. We found that ablation of antennae had no effect on oviposition avoidance to putrescine showing that IR41a/IR76b OSNs were dispensable once the female had closed in on an egg-laying substrate (Fig 3E, S4G Fig). We therefore turned to the other chemosensory modality, gustation. Taste organs are specified during development by the transcription factor Poxn. In *Poxn* mutants, most taste organs with the exception of some taste organs in the pharynx [53] are transformed into mechanosensory organs, including those found on the labellum, the legs, and the wings [54,55]. Compared to wild type females, the taste-impaired *Poxn* mutant females completely lost their aversion to oviposit onto polyamine-rich substrate and laid their eggs in equal numbers of both sites of the assay (Fig 3F, S4H Fig). This phenotype was rescued to wild type levels when *Poxn* was re-expressed using a full-genomic *Poxn* construct that rescued all taste neurons (Fig 3F, S4H Fig) [55]. Therefore, we concluded that the ultimate choice for egg-laying choice depends on GRNs, but does not involve pharyngeal taste neurons. We next sought to determine the external taste organs most critical for the female’s egg-laying decision. To this end, we ablated single taste organs and assayed oviposition behavior. Ablation of the lower segments of front, middle, or hind legs had no effect on the oviposition choice of wildtype females and the majority of eggs were laid on the polyamine-free site (Fig 3E, S4G Fig). Similarly, females with clipped wings behaved normally to polyamines (Fig 3E, S4G Fig). By contrast, ablation of the labellum resulted in equal egg-laying on both sides of the plate and complete loss of oviposition preference, strongly suggesting, together with the *Poxn* mutant data, that polyamine-based oviposition choice requires taste neurons on the fly’s labellum (Fig 3E, S4G Fig). As we cannot ablate all legs simultaneously, it remains possible that tarsal IR76b neurons also contribute to some extent to the choice. Nevertheless, these other IR76b neurons cannot compensate for the lack of labellar neurons. Thus, gustatory neurons on the labellum are essential to mediate the short-range polyamine choice behavior during egg-laying.

Taste sensilla contain several types of neurons including bitter-, salt-, and sugar-sensitive neurons [37]. How flies taste any amine including polyamines is unknown [37]. We thus sought to determine which taste receptors mediate the gustatory perception of polyamines. Some GRNs express IRs [37,56]. In contrast to the odor-detecting IR41a, IR76b is expressed in the gustatory system including GRNs of the labellum. These IR76b neurons project to the SEZ [40] (Fig 3G). Given the requirement of IR76b olfactory neurons in attraction to polyamines, we tested the involvement of their gustatory counterparts in oviposition behavior. Four loss of function mutants of IR76b (e.g., *IR76b*^*1*^), as well as the Gal4-mediated neuronal silencing of IR76b neurons (*IR76b-Gal4;UAS-Kir2*.*1*) completely abolished polyamine avoidance during egg-laying (Fig 3H, S4I Fig). By contrast, mutants for other IRs (e.g., *IR8a and IR25a*) or *ORCO* or silencing of sugar-sensitive neurons (GR5a and GR64f) by Kir2.1 had no effect on polyamine avoidance of pure polyamine-agar substrate during oviposition (Fig 3I, S4I–S4K Fig). These data implicate labellar IR76b taste neurons into the detection of polyamine taste.

Polyamines are strongly bitter-tasting compounds to humans [57]. We tested whether this sensation could trigger the aversion of polyamines in the absence of fruit or sugar (see above). When we silenced bitter-sensing GR66a taste neurons [58] during egg-laying, the aversive (pure) polyamine became attractive to these females (*GR66a-Gal4;UAS-Kir2*.*1*), which then strongly preferred to lay their eggs on the polyamine-rich otherwise plain substrate (Fig 3I, S4K Fig). These results confirm that polyamines are highly attractive egg-laying substrates and suggest that GR66a neurons may inhibit or counteract such attractiveness. This result also provides a mechanistic explanation for why polyamines in the context of fruit or sugar are highly attractive to flies, because sweet sensation can quench the perception of bitter ([59] see Fig 3D, S4E and S4F Fig). Silencing of both IR76b and GR66a neurons simultaneously (*GR66a-Gal4*,*IR76b-Gal4;UAS-Kir2*.*1*) abolished this preference for the polyamine-rich substrate. This further demonstrates that the attraction to polyamine indeed depends on IR76b (Fig 3I, S4K Fig).

Given that IR76b and GR66a are both expressed in taste organs, we carried out double-labeling experiments using genetic reporters to analyze their relative position on the labellum and their axonal projections into the SEZ (Fig 3G, S5 Fig). We first confirmed that *IR76b-QF* and *IR76b-Gal4* used in the behavioral assays were coexpressed in gustatory neurons. Although the relative expression levels of the two reporters showed some variation, they were by and large coexpressed (S2 Fig). We therefore used the *IR76b-QF* reporter to analyze potential expression in GR bitter neurons (*GR66a-Gal4*). Our reporter expression data suggested that these receptors are not expressed in the same neurons in the labellum as the respective axon populations innervated distinct regions of the SEZ (Fig 3G, S5 Fig, e.g., SEZ panel) [40]. These results suggest that two different taste neuron populations on the labellum are required to taste and evaluate polyamine-rich egg-laying substrates.

To strengthen this evidence and to confirm that IR76b receptors were indeed required to taste polyamines, we carried out rescue experiments in *IR76b*^*1*^ mutant females. Re-expression of IR76b in IR76b receptor neurons (*IR76b-Gal4*,*UAS-IR76b;IR76b*^*1*^) resulted in a full rescue of oviposition behavior (Fig 3J, S4L Fig). By contrast, re-expression of IR76b selectively in IR41a OSNs (*IR41a-Gal4*,*UAS-IR76b;IR76b*^*1*^) did not rescue oviposition choice confirming that this behavior depended on taste neurons (Fig 3J, S4L Fig). Finally, we re-expressed IR76b in GR66a neurons (*GR66a-Gal4*,*UAS-IR76b;IR76b*^*1*^*)* and observed no rescue of the choice behavior confirming that IR76b receptors critical for polyamine taste do not reside in GR66a bitter neurons (Fig 3J, S4L Fig). Hence, the fly receives and integrates two types of information, quality and valence, from one molecule, using two types of taste neurons. Given our data on polyamine and apple juice/sugar, such integration could potentially follow a mechanism that was recently demonstrated for sugar neurons, which are indirectly inhibited by bitter neurons via GABAergic inhibitory neurons of the SEZ [59]. This type of multimodal taste integration would allow the fly to measure the relative levels of polyamines and sugars during the evaluation of food.

### Taste Neurons Respond to Polyamines

**Fig 4:**
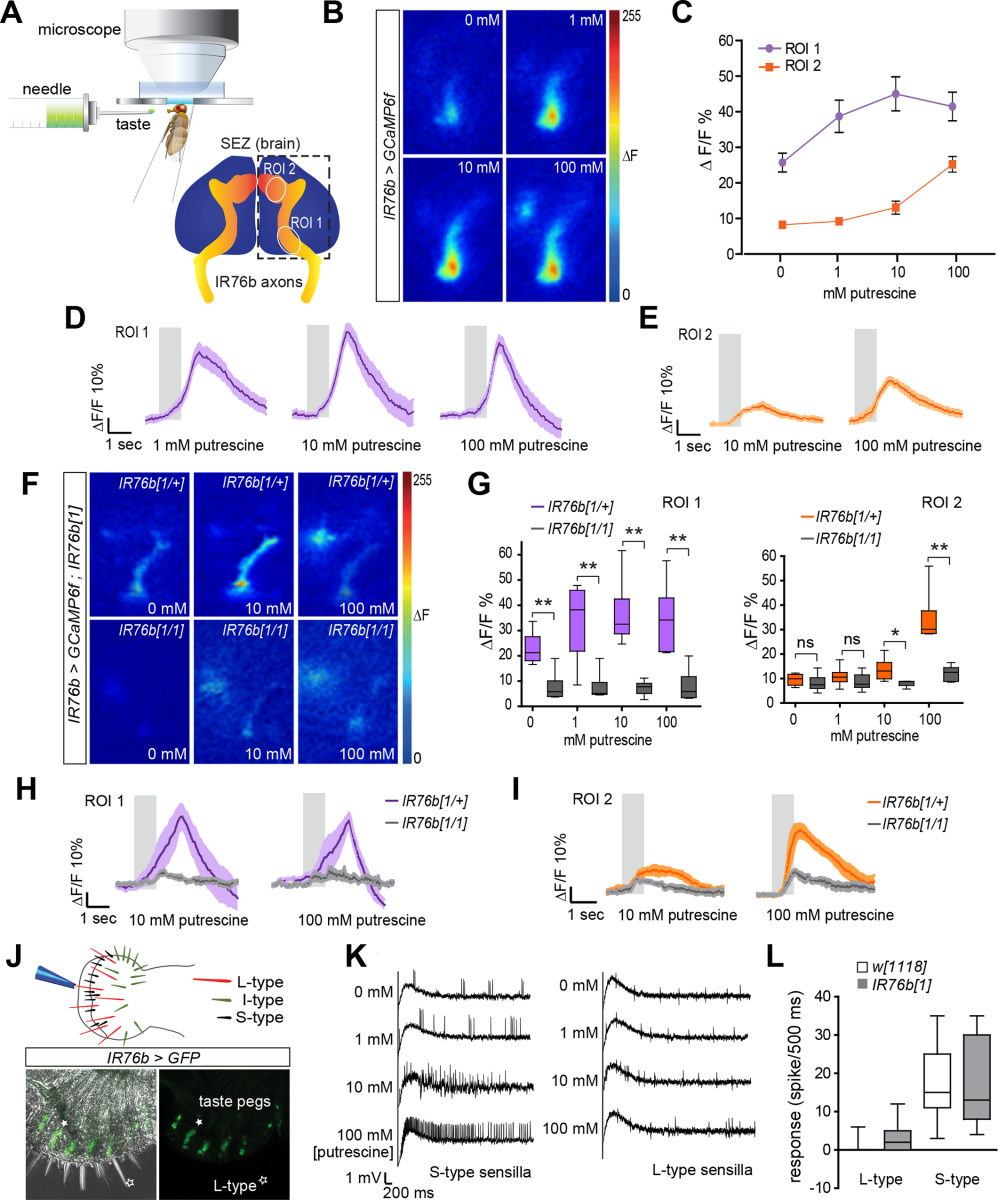
IR76b neurons respond to polyamines in gustatory processing. (A) Schematic drawing of SEZ calcium imaging setup and position of ROIs within the SEZ, which were used for quantification of relative changes in GCaMP-fluorescence (%ΔF/F). (B) Representative images of SEZ imaging of *IR76b-Gal4;UAS-GCaMP6f* female flies stimulated with distilled water or increasing concentrations of putrescine. (C) Quantification of GCaMP6f-fluorescence peak responses (in %ΔF/F) in the ROI 1 and ROI 2 area, respectively, when female flies were stimulated with increasing concentrations of putrescine (*n* = 11 ± SEM). (D) Average response trace of ROI 1 area to putrescine (*n* = 11 ± SEM). The gray bar shows the stimulation period. Dark colored line in the middle presents the average value, and the light shade presents the SEM. (E) Average response trace of ROI 2 to putrescine (*n* = 11 ± SEM). (F) Representative images of SEZ of *IR76b-Gal4*,*UAS-GCaMP6f; IR76b*^*1*^ and heterozygous control female flies stimulated with distilled water or increasing concentrations of putrescine. (G) Quantification of peak responses (in %ΔF/F) in *IR76b* mutant and control. Responses in the ROI 1 and ROI 2 are calculated separately with increasing concentrations of putrescine (*n* = 6 ± SEM). Boxes show median and upper/lower quartiles, and whiskers show minimum/maximum values. (H) Average response trace of ROI 1 in *IR76b* mutant and control (*n* = 6 ± SEM). (I) Average response trace of ROI 2 in *IR76b* mutant and control (*n* = 6 ± SEM). (J) Scheme of the *Drosophila* labellum with different types of sensilla. IR76b *(IR76b-Gal4;UASmCD8GFP)* is expressed in peg taste sensilla on the labellum. Filled star indicates peg taste neuron, open star indicates L-type sensillum. (K) Electrophysiological recording of S-type and L-type sensilla to putrescine at different concentrations (0 mM–100 mM, *n* = 8 ± SEM). (L) Quantification of the response of S-type and L-type sensilla to putrescine in *IR76b* mutant and wild-type control. (*n* = 8 ± SEM). All *p*-values were calculated via Student’s *t* test (ns > 0.05, *p ≤ 0.05, **p ≤ 0.01, ***p ≤ 0.001).

Our behavioral data provides strong evidence that polyamine sensing in the context of oviposition requires IR76b receptor in taste neurons on the female’s labellum. To test more directly whether taste neurons responded to polyamines, we monitored the response of IR76b neurons to putrescine using in vivo calcium imaging with GCaMP6f (*IR76b-Gal4; UAS-GCaMP6f*; Fig 4A). Labellar stimulation with a putrescine solution led to a significant increase in GCaMP-fluorescence in primarily two regions of the SEZ innervated by at least two distinct sets of IR76b taste neurons (Fig 4A–4E). One of these regions (region of interest [ROI] 1) responded more strongly at for egg-laying behavior relevant concentrations (1 mM) (Fig 4C and 4D). By contrast, ROI 2 responded to higher concentrations of polyamine (100 mM; Fig 4C and 4E). We also observed calcium increases in ROI 1 in IR76b neuron axon terminals upon stimulation with salt consistent with a previous report implicating IR76b in the detection of low salt concentrations (S6A Fig, 50 mM NaCl, [40]). Given that IR76b neurons are also found on the legs, we tested their responses to polyamines. To this end, we recorded GCaMP-fluorescence directly from the neurons on the tarsae (S6B–S6D Fig). Our results suggest that polyamine-sensitive tarsal taste neurons exist on all legs and therefore could potentially contribute—although probably to a lesser extent—to the egg-laying choice. Of note, we only observed a significant response to high (100 mM) but not to lower polyamine concentrations (1 mM).

To gain more evidence for a role of IR76b as polyamine receptor, we recorded changes in GCaMP fluorescence in *IR76b*^*1*^ mutant flies and found that the response to polyamine was absent in both the ROI 1 and ROI 2 areas (Fig 4F–4I). Similarly, the response to salt was also significantly reduced (S6E Fig). These results demonstrate that IR76b receptor is required for polyamine detection by GRNs. Together with our behavioral data, these results show that IR76b, in addition to its function as salt receptor [40], is a taste receptor for polyamines.

We wondered which type of IR76b-expressing labellar GRN detects polyamines. Labellar taste neurons are housed in three types of taste bristles, short (S), intermediate (I), and long (L), as well as in taste pegs [35] (Fig 4J). L-type GRNs expressing IR76b promote the attraction to low levels of salt [40]; and IR76b expression as judged by the Gal4 reporter (*IR76b-Gal4;UAS-mCD8GFP*) can be observed in L-type sensilla [40]. In addition, we found that the reporter was strongly expressed in taste pegs (Fig 4J). Consistent with this, the innervation of the ROI 1 area in the SEZ by IR76b GRN axons resembled the innervation previously observed for GRNs in taste pegs (e.g., GRNs responding to carbonation, [60]). As the ROI 1 area responded most strongly to behaviorally relevant concentrations of polyamines, it is conceivable that IR76b peg neurons are required for oviposition choice behavior. To test which neurons responded to the taste, we used tip recordings and recorded putative responses of the taste neurons housed in sensilla. In particular, we asked whether L-type sensilla were activated by polyamines. We found that none of the recorded L-type sensilla showed a response to putrescine consistent with our hypothesis that a different GRN type detects polyamines such as the peg neurons (Fig 4K and 4L). We next tested the responses of S-type sensilla. These sensilla responded significantly to polyamines (Fig 4K and 4L), but IR76b did not mediate these responses, as tip recordings on *IR76b*^*1*^ mutants still showed strong responses of S-type sensilla to putrescine (Fig 4L). Given that bitter receptors are prominent in these sensilla, we hypothesize that bitter receptors such as GR66a might instead mediate these responses consistent with their involvement in oviposition behavior.

We conclude that the fly’s taste sensation of polyamines requires IR76b in labellar GRNs, most likely in peg neurons but not in L-type sensilla that mediate the response to salt [40]. IR76b is also not required in S-type sensilla, which presumably mediate the bitter taste of these compounds. Finally, it appears that amongst IR76b taste neurons, some are involved in the sensation of polyamines while others sense salt, indicating the involvement of up-to-now unidentified partner receptors for salt and polyamines, respectively.

### Multisensory Detection of Polyamines Explain Egg-Laying Behavior

**Fig 5:**
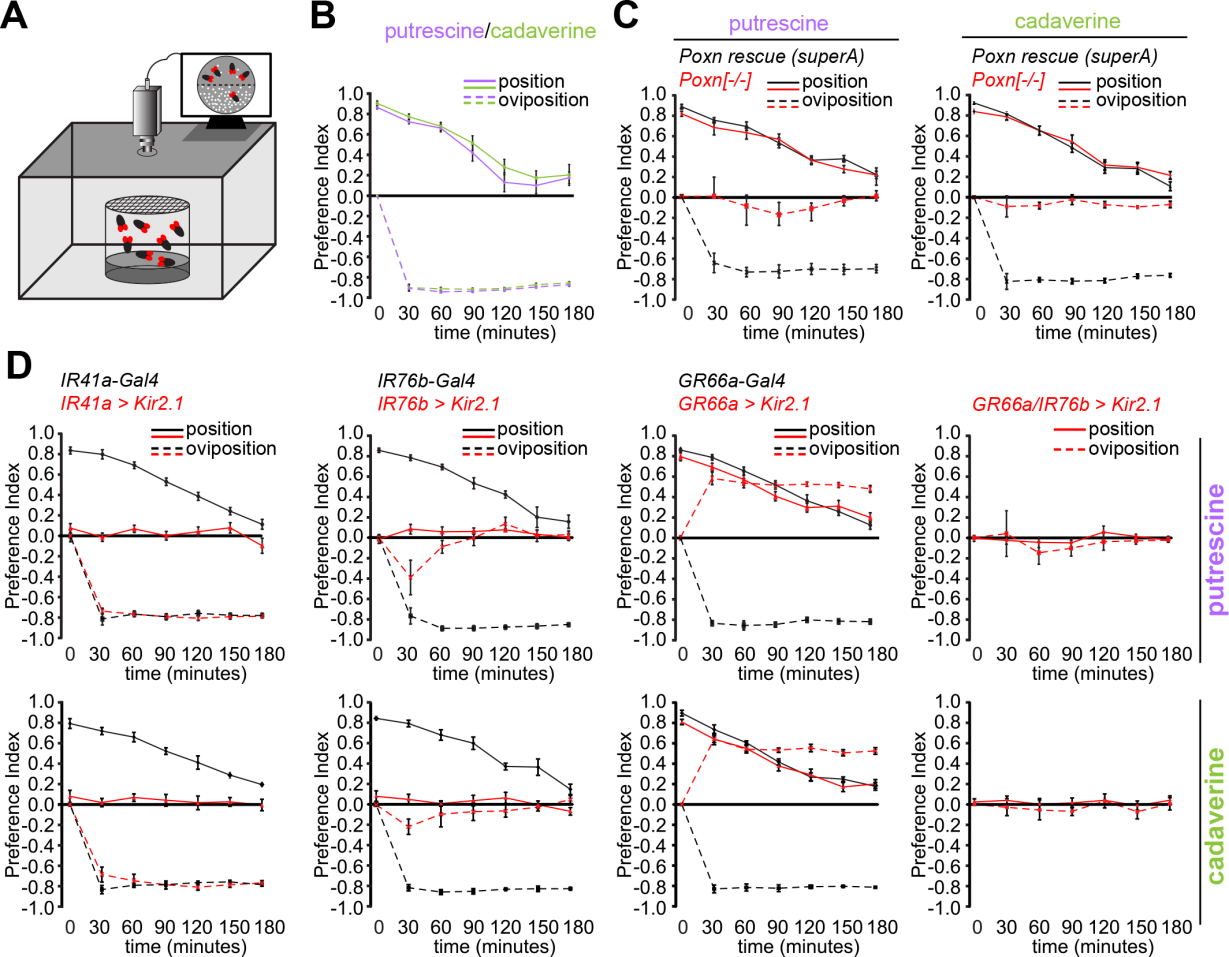
Females detect odor and taste of polyamines during oviposition site selection. (A) Scheme of the video-tracking position-oviposition assay. (B) Females spend more time on the polyamine-rich site but avoid pure polyamines for egg laying. Position (solid line) and oviposition (dashed line) preference of Canton S to putrescine and cadaverine over time in position–oviposition assay. *y*-axis shows position and oviposition PI. *x*-axis shows total time (min) of the assay. The female’s position–oviposition behavior was quantified in 30 min intervals. Box plots show median and upper/lower quartiles (*n* = 8, 60 ♀ flies/trial, total time of assay of 3 h. (C) Position and oviposition preference of loss of function mutant of *Poxn* (*Poxn*^*-/-*^) and *Poxn* full rescue (rescue superA) to putrescine in position–oviposition assay. Red lines indicate behavior of *Poxn*^*-/-*^ females, while black lines designate behavior of *Poxn* rescue SuperA. Loss of sense of taste does affect oviposition but not position preference. Box plots show median and upper/lower quartiles (*n* = 8, 60 ♀ flies/trial). (D) Position and oviposition preference of *IR41a-Gal4;UAS-Kir2*.*1*, *IR76b-Gal4;UAS-Kir2*.*1*, *Gr66a-Gal4;UAS-Kir2*.*1*, and *Gr66a-Gal4*,*IR76b-Gal4;UAS-Kir2*.*1* and their respective genetic controls to putrescine over time in position–oviposition assay. Position preference is mediated by IR41a olfactory neurons, while taste neurons trigger oviposition avoidance. Red lines indicate *receptor-Gal4;UAS-Kir2*.*1*, while black lines indicate controls. Box plots show median and upper/lower quartiles (*n* = 8, 60 ♀ flies/trial). All *p*-values were calculated via two-way ANOVA with the Bonferroni multiple comparison posthoc test (ns > 0.05, *p ≤ 0.05, **p ≤ 0.01, ***p ≤ 0.001).

Our data suggest that female flies sense and interpret polyamine odor and taste. To address how flies use and integrate this multisensory input elicited by a single compound during egg-laying site selection, we video-monitored the time flies spend on polyamine-rich compared to control substrate in the setup of the oviposition assay (Fig 5A). To simplify the assay and the interpretation, we carried out the experiments on polyamine-rich substrates devoid of additional odors or tastes. We found that wild type female flies spent significantly more time on the polyamine site (positive position index; solid lines), although they preferred to lay their eggs on the control site (negative oviposition index; dashed lines) (Fig 5B, S7A Fig). Notably, flies showed the strongest positional preference for polyamine at the beginning of the experiment, but this preference steadily declined in parallel to an increase of eggs laid (Fig 5B). We investigated which of the identified receptor neurons and thus sensory modalities are used for position and oviposition, respectively. Taste neuron-deficient *Poxn* mutants confirmed that oviposition aversion was dependent on taste; *Poxn* mutants showed no aversion and laid their eggs on both sides of the assay (Fig 5C, S7B and S7C Fig). By contrast, positional attraction to polyamines remained intact in these flies (Fig 5C). Of note, *Poxn* mutant females laid significantly less eggs in the first three hours that were observed in this assay (S7B Fig). However, in the longer assay as shown above (S4H Fig), the total number of eggs was comparable to controls suggesting that a lack of the sense of taste may initially inhibit the female’s willingness to deposit her eggs.

We found that silencing IR41a OSNs (*IR41a-Gal4;UAS-Kir2*.*1*) selectively affected position preference and not oviposition avoidance, suggesting that positional preference required olfactory detection of polyamines (Fig 5D, S7D Fig). Furthermore, females with silenced IR76b neurons (*IR76b-Gal4;UAS-Kir2*.*1*), which included the IR41a/IR76b OSN population as well as the IR76b taste neurons, were completely indifferent to polyamines in position and oviposition (Fig 5D, S7D Fig). They also showed a similar phenotype as *Poxn* mutant females and appeared to hold their eggs longer before deciding to lay them consistent with a deficient taste system (S7D Fig and S4K Fig). Silencing of GR66a bitter taste neurons (*GR66a-Gal4;UAS-Kir2*.*1*) had no effect on position behavior, but as reported above, reversed oviposition avoidance to preference (Fig 5D, S7D Fig). Egg numbers were comparable to controls (S7D Fig). Furthermore, concurrent inactivation of IR76b and bitter tasting neurons (*Gr66a-Gal4*,*IR76b-Gal4;UAS-Kir2*.*1*) completely abolished positional and oviposition preference (Fig 5D, S7D Fig).

In summary, these data show that odor- and taste-induced behaviors do not depend on each other but rather happen in a parallel or sequential manner. Polyamine odors could be long-range attractive cues for female flies to navigate to putative oviposition sites. Upon arrival on or in close proximity of such sites (a decaying fruit or the confined space of our oviposition assay), the sense of smell is dispensable, and females refine their choice for oviposition with their taste organs, likely integrating polyamine inputs with other tastes (e.g., sugar).

### Polyamines Attract *Ae*. *aegypti* Mosquitoes to Egg-Laying Sites

Having shown that *Drosophila* flies use polyamines to identify egg-laying sites, we next investigated a more general role of these compounds beyond the vinegar fly. *Ae*. *aegypti* mosquitoes transmit the dangerous disease dengue fever and cause about 25,000 deaths per year [61]. ORs are among the potential targets of measures for pest control.

**Fig 6:**
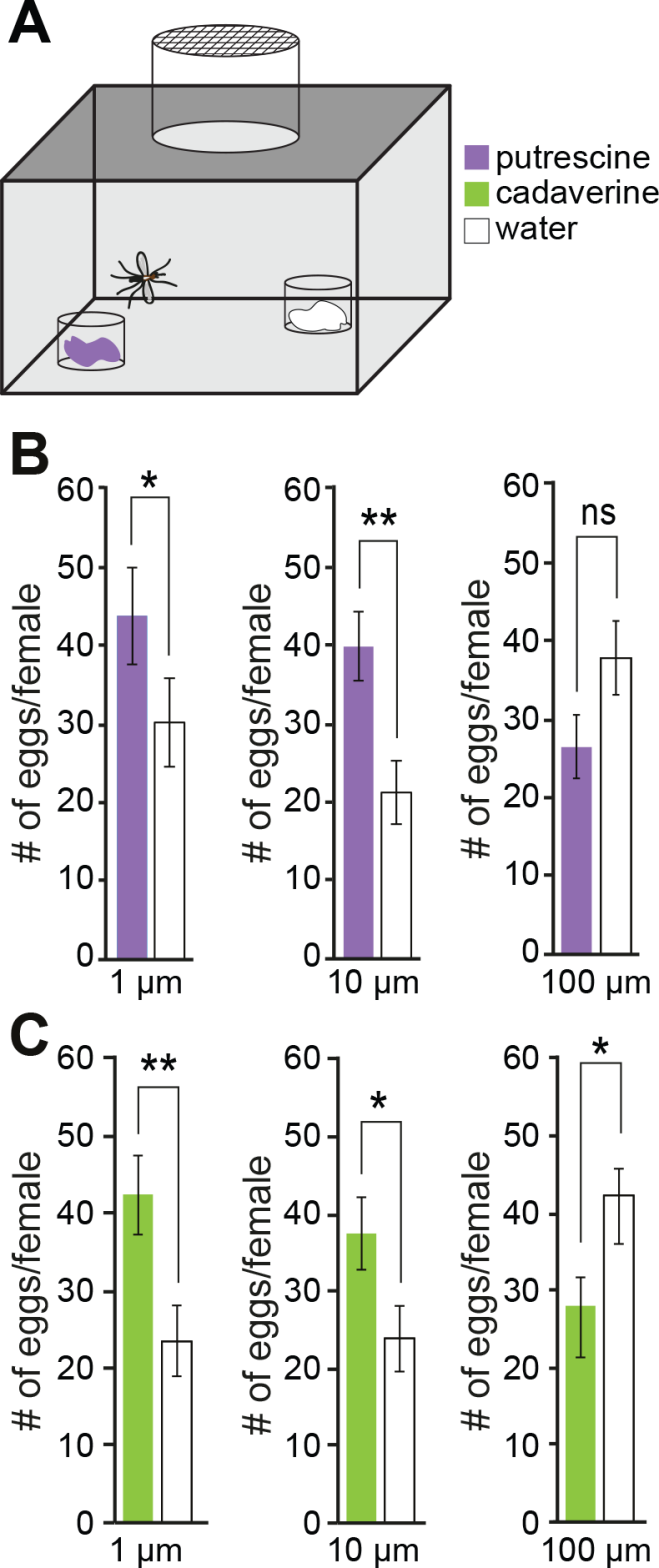
*Ae*. *aegypti* mosquitoes are attracted to polyamines for egg laying. (A) Schema of single female mosquito egg-laying assay. Mated, gravid females were given the choice between two small cups containing either pure water or water enriched with different concentrations of polyamines (1 μM to 100 μM). Females were exposed only to the odor of polyamines, and direct contact to polyamines was prevented (see materials and methods). (B–C) Females were most attracted to 1 and 10 μM of putrescine (purple, upper panels) or cadaverine (green, lower panels). 100 μM polyamine repelled females from laying eggs into the cup. PIs are averaged (*n* = 8 ± SEM, 1 female/trial). All *p*-values were calculated via Student’s *t* test (ns > 0.05, *p ≤ 0.05, **p ≤ 0.01).

Because *Aedes* mosquito adults live in human households and are often found attached to sheets, curtains, etc. [62], insecticide treatments of adults are often limited by their proximity to humans. Targeting breeding sites might therefore be more practical and efficient [63]. Using laboratory oviposition assays, we asked whether *Aedes* females are attracted to the odor of polyamines for egg-laying (Fig 6A). Single females were released into a caged area and given the choice between laying into a cup that smelled of polyamines and an odorless cup. They were prevented from tasting the compounds in this assay and forced to use their sense of smell. We found that egg-laying female mosquitoes deposit significantly more eggs into water that smelled of putrescine or cadaverine compared to control (Fig 6B and 6C). This attraction was concentration-dependent, and mosquitoes were most attracted at 1μM and 10 μM of putrescine or cadaverine (Fig 6B and 6C). By contrast, concentrations as of 100 μM started to become repulsive, and females preferred to lay their eggs into the nonsmelling cup (Fig 6B and 6C).

These experiments suggest that also other insects such as mosquitoes use polyamines to find feeding and egg-laying sites. These polyamine-based behaviors and possibly the detection mechanisms might be conserved in other species.

## Discussion

Here, we show that polyamines, important biogenic amines, are beneficial components of a diet that increases the reproductive success of *Drosophila* flies. In line with this, flies are highly attracted to polyamines and use them to identify food sites for instance for egg-laying. The decision to approach or lay eggs on polyamine-rich substrates requires multisensory integration of two distinct sensory modalities, olfaction and taste. We have identified the sensory and molecular mechanisms involved in polyamine-guided behavior in both modalities and show that they share the requirement of the IR, IR76b.

### Polyamines Are Beneficial Nutrients Involved in Reproductive Success

Polyamines represent an important component of animal nutrition [6,12]. Deficiency as well as excess of polyamines can be detrimental to health and reproduction [6]. Therefore, species might have undergone a selection to choose food with levels of polyamines that meet their physiological needs. Our results provide important biological significance for the preference of *D*. *melanogaster* for polyamine-rich food such as fermented fruit or fresh oranges (see also [14,15]). In particular, we show that a polyamine-enriched diet increases the number of offspring produced by a fly couple. Females feeding on polyamine-supplemented food lay significantly more eggs. Mechanistically, we speculate that the diverse and conserved roles of polyamines during cell cycle progression, differentiation, and autophagy among others are responsible for the beneficial effect [5]. Interestingly, in addition to uptake through the diet, polyamine-synthesis enzymes (i.e., ornithine decarboxylase) are selectively up-regulated after mating in the spermatheca of female mosquitoes, a tissue involved in sperm storage, egg-production, and laying in mosquito and *Drosophila*, consistent with a role in fertility and reproduction [64].

Conception and proper embryonic development depend on polyamines also in humans [5]. Polyamines also form a substantial part of the male ejaculate (i.e., spermine and spermidine) and infertile men show lower levels of spermine, spermidine, and putrescine in their semen [5]. Interestingly, polyamine-synthesizing enzymes in the cell decay with age, spurring studies on the beneficial effects of polyamines in the diet. Polyamine supplementation might counteract age-related loss of fertility in men and women, but also other ageing-induced deficits such as loss of memory or even lifespan [4,8,10,11]. On the other hand, an excess of these compounds in the cell, and therefore possibly in the diet has been linked with the occurrence and progression of cancer and other diseases suggesting that polyamine intake should be carefully regulated [6]. With the characterization of sensory mechanisms underpinning the attraction to polyamines, we can begin to analyze how fundamental physiological needs shape sensory processing and ultimately impinge on feeding and reproductive behavior.

### Multisensory Detection of Polyamines

Flies detect the odor and taste of polyamines. In addition, our data suggests that two types of gustatory neurons evaluate quality and valence of polyamines separately. The female to identify egg-laying sites uses these two taste modalities, polyamine taste and bitter taste. In this context and presumably during feeding, sugar in the fruit appear to override the bitter taste of polyamines, which translates at the behavioral level to a preference for polyamine-rich compared to polyamine-poor fruity substrates. This claim is supported by data showing that female flies strongly prefer to lay their eggs into pure polyamine substrates when their bitter sense is silenced genetically. Therefore, several modalities contribute to polyamine choice behavior. Such multimodal detection of polyamines might ensure that the animal only consumes polyamines in the context of a suitable food source and at beneficial concentrations. A similar interaction was suggested between sugar and bitter taste sensation in the fly, indicating that concentrations might be generally estimated by assessing the relative amounts of tastants [59,65,66]. Multimodal taste experiences are essential to judge food quality also for humans. Therefore, sugar is frequently used to quench bitter tastes in food and to make medication more palatable [67].

In addition to integrating two types of taste modalities, flies also appear to use their sense of smell to find polyamine-rich foods. Our tracking data shows that these two kinds of information, smell and taste, are used sequentially consistent with odor being a long-range and taste a short-range signal. Flies that have found the source of the polyamine using their sense of smell do no longer require it to make the decision on where to lay eggs. However, whether odor and tastes are integrated in a more complex odor/taste environment of polyamines and other cues remains to be investigated.

### IR76b Receptors Are Required to Detect the Smell and Taste of Polyamines

Our behavioral data shows that flies are attracted to all polyamines tested including putrescine, spermine, and cadaverine. Notably, they appear to prefer concentrations that are typically found in fermented foods, overripe fruit, or oranges, also a favorite of flies [15]. The receptor IR76b is required for the detection of the odor as well as the taste. Mutants for *IR76b* show significantly reduced attraction to polyamines. In this context, IR76b seems to work with another receptor, IR41a, which is specific to a very small subset (~7) of antennal OSNs. Although IR76b is more broadly expressed than IR41a in the olfactory system, it is unclear whether it plays the role of a coreceptor like IR8a or IR25a [31]. IR25a, which appears to be coexpressed with IR76b and IR41a, could play this role in polyamine detection. However, *IR25a* mutant flies show no decrease in polyamine attraction compared to controls. Thus, it is possible that IR76b and IR41a work as functional receptor heteromers, a configuration that is necessary and sufficient to form functional chemoreceptors [30,31].

Notably, the effect of the *IR76b* mutation in calcium imaging of OSNs appeared significantly stronger than the effect of the mutation in odor-guided behavior (see Fig 1 and Fig 2), although IR76b and the IR41a glomerulus appeared to be necessary and sufficient to mediate polyamine odor attraction (see Fig 1). This difference could be due to the slightly different conditions in behavior and imaging. On the one hand, animals are freely moving during behavior and might experience odor plumes rather than constant streams. On the other hand, animals are exposed for longer periods of times to the odor in the T-maze as compared to the imaging experiments. These effects might also explain a discrepancy between our behavioral data and a previous study implicating the IR coreceptor IR8a in the detection of putrescine using single sensillum electrophysiology [31]. As mentioned above the same *IR8a* loss of function mutants as used by Abuin et al. [31] did not show any significant reduction in polyamine odor attraction in behavioral assays.

In the gustatory system, IR76b expressed in labellar GRNs is necessary and sufficient to mediate polyamine choice behavior. In contrast to the olfactory system, a mutation in *IR76b* results in complete loss of preference behavior to polyamines as well as a loss of calcium responses in GRNs. Calcium imaging and tip recording along with expression data indicate that polyamines are not recognized by IR76b expressed in L-type or S-type sensilla on the labellum, but instead might be detected by peg taste neurons that express IR76b. Furthermore, IR76b taste neurons on the leg, although not essential for egg-laying decisions, responded to polyamines. Based on our data, we can exclude and infer the involvement of certain types of IR76b taste neurons for polyamine detection, but further experiments will be required to reveal their exact identity and position.

A previous study showed that IR76b in L-type neurons was required for the fly’s attraction to low levels of salt [40]. How can the same receptor mediate two or more different taste modalities? The easiest explanation might be the involvement of at least one coreceptor for either salt or polyamine. Given that IR76b expressing L-type sensilla do not respond to polyamines, this appears to be a likely scenario at least for the detection of polyamines. Up to now, our candidate approaches, however, have not identified such a coreceptor. The other possibility is that the same receptor has putative binding sites for both of these ligands. In fact, ORs with few exceptions such as the CO_2_ receptors [24,26] detect multiple odorants. Nevertheless, salt and polyamines appear to be very different types of ligands. The activation of IR76b receptor by salt seems to depend on a particular amino acid located in the transmembrane domain, which is required for ion conductance in ionotropic glutamate receptors (iGluRs) [40]. The authors propose that this amino acid will keep the channel in a constitutively open or partly open position, which allows sodium entry when GRNs contact salt.

The structural similarity of IRs and iGluRs might provide hints for how polyamines could activate IRs. Polyamines are released from presynaptic terminals and can thus interact with the extracellular domains of synaptic iGluRs [68]. Such interactions appear to modulate the activity of some iGluRs (e.g., [69]). For instance, spermine can potentiate the activity of NMDA (N-methyl-D-aspartate) receptor by binding at a site within the extracellular domains of one of the subunits, the NR1 subunit [69–71]. Comparison of the putative structures of IR41a and IR76b and the structure of the NR1 subunit shows that these receptors share a high structural similarity (S8 Fig). It is thus conceivable that polyamine activation of IR41a and IR76b follows a similar mechanism as polyamine potentiation of NMDAR [72]. Structure–function analysis guided by studies on the NMDAR will help to test this model. It is certainly exciting to speculate that the binding and modulation by polyamines has been acquired early on in the evolution of iGluRs and further optimized in specific IRs.

## Materials and Methods

### Fly Rearing and Lines

*D*. *melanogaster* stocks were raised on conventional cornmeal-agar medium at 25°C temperature and 60% humidity and a 12 hr light:12 hr dark cycle. Following fly lines were used to obtain experimental groups of flies in the different experiments:

(1) Canton S
(2) *w*^*1118*^
(3) *orco*^*1*^
(4) *eyflp; FRT82B CL / FRT82B*
(5) *eyflp; FRT82B CL / FRT82B ato*^*1*^
(6) *IR8a*^*1*^*;Bl*^*1*^ *L*^*2*^*/CyO*
(7) w*; IR25a^*1*^*/CyO*
(8) w*; IR25a^*2*^*/CyO*
(9) *y*^*1*^w*;P(w[+mC] = UAST-YFP.*Rab39*.*S23N]IR76b*^*05*^
(10) *y*^*1*^*w[67c23];Mi[ET1]IR76b [MB00216]*
(11) w*;IR76b^*1*^
(12) w*;IR76b^*2*^
(13) w*;+; UAS-Kir2.*1*::*eGFP*
(14) w*;P[IR41a-GAL4.*2474]attP40;TM2/TM6B*,*Tb*
(15) w*; P[IR76b-GAL4.*916]226*.*8;TM2/TM6B*,*Tb*
(16) w*;P[IR76b-QF.*1*.*5]2*
(17) *w*^*1118*^*;UAS-mCD8GFP*,*QUAS-mtd-tomato-3xHA*
(18) *w*^*1118*^*;UAS-IR41a-RNAi*:*P[KK104134]VIE-260B (a)* and
*y*^*1*^*v*^*1*^*; P[y[+t7*.*7] v[+t1*.*8] = TRiP*.*HMJ21838]attP40 (b)*
(19) *w*^*1118*^*;Poxn[ΔM22-B5]/CyO*, (a genomic deletion of the *Poxn* locus; a detailed description of the allele can be found in [73])
(20) *w*^*1118*^*;Poxn[ΔM22-B5];P[w6 Poxn_resc]superA158-119*, (the same genomic deletion of the *Poxn* locus in combination with a genomic rescue construct that rescues all aspects of *Poxn* loss of function; a detailed description of the allele can be found in [73])
(21) w*;GR5a-Gal4-6/CyO
(22) w*;GR64f-Gal4/CyO
(23) w*;GR66a-Gal4/Cyo;TM2/TM6B, *Tb*
(24) *w*^*1118*^*; PBac[w[+mC] = WH]IR31a[f06333]*
(25) *y*^*1*^ *w[67c23]; Mi[ET1]IR75a[MB00253]*
(26) *y*^*1*^w*; Mi[y[+mDint2] = MIC]IR75a[MI00303]
(27) *w*^*1118*^*;Mi[ET1]IR75d[MB04616]*
(28) *y*^*1*^w*;Mi[y [+mDint2] = MIC]IR84a[MI00501]
(29) *w*^*1118*^*; Mi[ET1]IR92a[MB03705]*
(30) w*;UAS-mCD8GFP
(31) w*;UAS-GCaMP6f
(32) w*; Bl^*1*^*/CyO; P[w[+mC] = UAS-Ir76b*.*A]298*.*7* and
w*; P[w[+mC] = UAS-Ir76b.*Z]2/CyO; TM2/TM6B*, *Tb*

The majority of the lines were obtained from Bloomington (http://flystocks.bio.indiana.edu/) or the VDRC stock center (http://stockcenter.vdrc.at). The Poxn lines were a gift by Werner Boll and IR76b-QF was a gift by Craig Montell.

### Behavioral Assays for *Drosophila melanogaster*

#### T-maze assay

The use of the T-maze assay is indicated in all figures with a fly head schematic with red colored antennae to show that polyamine preference depends on OSNs on the antenna. Five to seven days old flies raised at 25°C were used for all experiments with the exception of experiments where RNAi was used. For these experiments, flies were raised at 30°C to increase the efficacy of RNAi. Flies were tested in groups of ~60 (30 females and 30 males or 60 females) in a T-maze and were allowed 1 min to respond to stimuli. Experimentation was carried out within climate controlled boxes at 25°C and 60% rH in the dark. 50 μl of fresh odor solution at different concentrations diluted in distilled water applied on Whatman chromatography paper was provided in the odor tube while 50 μl of distilled water (polyamine solvent) applied on Whatman chromatography paper was placed into the control tube. Unless otherwise indicated 1 mM (according to photo-ionization detector (PID) measurements corresponds to ~10 ppm) of either putrescine or cadaverine were used. After experimentation, the number of flies in each tube was counted. An olfactory PI was calculated by subtracting the number of flies on the test odor site from the number of flies on the control site and normalizing by the total number of flies. Statistical analysis was performed using two-way ANOVA and the Bonferroni multiple comparisons post-hoc test using Prism GraphPad 6.

#### Oviposition assay

The oviposition assay is indicated in all figures by an illustration of the fly head with a red-labeled proboscis showing that oviposition preference depends on labellar taste neurons. In addition, oviposition assays are shown in simple schemes in most figures. Here, the gray circle shows the oviposition plate filled with 1% agarose and the colored squares indicate the addition of putrescine or cadaverine on one half of the plate. Unless otherwise stated 1 mM of polyamine was used in all assays. Five to seven day old flies raised at 25°C were used for all experiments with the exception of experiments where RNAi was used. For these experiments, flies were raised at 30°C to increase the efficacy of RNAi. Mated female flies, reared on standard cornmeal medium at 25°C and 60% rH, were separated on ice from male flies at day 4 d posteclosion. Female flies were kept for two more days on fly food and used on day 6 for the oviposition assays. 1% low melting agarose was poured in 60 x 15 mm petri dish, and two halves were marked with a permanent marker on the bottom of the dish. 50 μl of polyamine solution at different concentrations was applied on one site (test) of the dish. In initial experiments, we also tested odor mixed into 1% low melting agarose compared to agarose only and obtained the same results as with applying the polyamine solution onto the hardened agarose. 60 female flies were put in a gauzed top round cage and the cage was closed with the test petri dish. Flies were kept for exactly 16 h in a light:dark cycle at controlled temperature and humidity conditions. An oviposition PI was calculated by subtracting the number of eggs on the test site from the number of eggs on the control site and normalized by the total number of eggs. Statistical analysis was performed using two-way ANOVA and the Bonferroni multiple comparisons posthoc test using Prism GraphPad 6.

#### Position-oviposition assay

The same experimental set up as used for the regular oviposition assays was placed in 75 x 45 x 47 cm black box with infrared light at 25°C and 60% rH for 3 h. Behavior of flies was video-tracked, and position preference of flies was quantified in 30 min time intervals. The number of eggs was quantified also every 30 min for 3 h. Position and oviposition preference indices were calculated as described above for individual time points. Statistical analysis was performed using standard two-way ANOVA and the Bonferroni multiple comparisons post-hoc test using Prism GraphPad 6.

### Behavioral Assays for *Ae*. *aegypti*

#### Animal rearing

*Ae*. *aegypti* (Rockefeller strain) were reared at 27 ± 2°C, 70 ± 2% RH under a 12 h:12 h light:dark period. Larvae were reared in plastic containers (20 x 18 x 7 cm) and fed Superwhite fishfood (Tropical, Poland). Pupae were transferred into 20 ml plastic cups and placed into Bugdorm-1 cages (Megaview, Taiwan; 30 x 30 x 30 cm). Adults had ad libitum access to 10% sucrose presented on a filter paper. Four to six days post emergence, females were starved for 6 h prior being offered defibrillated sheep blood (Håtuna lab, Sweden) through a membrane feeding system (Hemotek Ltd, UK). After taking a full blood meal, the females were transferred to the experimental chamber for oviposition experiments. Females had ad libitum access to the sucrose solution.

#### Oviposition assay

Oviposition assays were performed in a climate chamber maintained under the same conditions as the rearing chamber. The assays consisted of a Bugdorm-1 cage with two oviposition cups diagonally oriented at a distance of 20 cm from each other. Four separate cups made up an oviposition cup. In the bottom, a transparent 250 ml cup (Houseware Nordic Business Association AB, Sweden), filled with 1 ml of the test compound or control (water). Two paper cups (230 ml; Clas Ohlson, Sweden), the first with a 10 mm centre hole and the second with eight 1 mm piercings, were stacked into the plastic cup. In the second paper cup, a 30 ml cup (Nolato Hertila, Sweden), with a folded filter paper (Munktell Filter AB, Sweden) and 5 ml dH2O, provided the oviposition substrate. Four days post blood meal, female mosquitoes were transferred from the rearing cage to individual test cages where they were deprived of the sucrose solution. Female *Ae*. *aegypti* were then allowed to oviposit for three consecutive L:D cycles after which the number of eggs laid in the test cup and control cup were counted. The test compounds, putrescine and cadaverine, tested at 1, 10, and 100 μM, and the control, were refreshed on a daily basis. Statistical analysis was performed using Minitab (Minitab Inc, State College, Pennsylvania, USA).

#### Anatomy

Adult fly brains were dissected, fixed and stained as described previously [74]. Briefly, brains were dissected in cold PBS, fixed with paraformaldehyde (2%, overnight at 4°C or for 2 h at RT), washed in PBS, 0.1% Triton X-100, 10% donkey serum, and stained overnight at 4°C or for 2 h at RT with the primary and after washes in PBS, 0.1% Triton X-100 with the secondary antibody using the same conditions. All microscopic observations were made at an Olympus FV-1000 confocal microscope. Images were processed using ImageJ and Photoshop. The following antibodies were used: chicken anti-GFP (molecular probes, 1:100), rabbit anti-Dsred (Clontech, Living colors DsRed polyclonal AB, 1:200), and rat anti-N-cadherin (anti-N-cad DN-Ex #8, Developmental Studies Hybridoma Bank, 1:100). Secondary antibodies used were: anti-chicken Alexa 488 (molecular probes, 1:250) and anti-rabbit Alexa 549 (molecular probes, 1:250), respectively.

#### In vivo calcium imaging

For calcium imaging experiments, GCaMP6f was expressed under the control of IR41a-Gal4 or IR76b-Gal4. In vivo preparations of flies were prepared according to a method previously described [74]. In vivo preparations were imaged using a Leica DM6000FS fluorescent microscope equipped with a 40x water immersion objective and a Leica DFC360 FX fluorescent camera. All images were acquired with the Leica LAS AF E6000 image acquisition suit. Images were acquired for 20 s at a rate of 20 frames per s with 4 x 4 binning mode. During all measurements the exposure time was kept constant at 20 ms. For all experiments with odor stimulation, the stimulus was applied 5 s after the start of each measurement. A continuous and humidified airstream (2000 ml/min) was delivered to the fly throughout the experiment via an 8 mm diameter glass tube positioned 10 mm away from the preparation. A custom-made odor delivery system (Smartec, Martinsried), consisting of mass flow controllers (MFCs) and solenoid valves, was used for delivering a continuous airstream and stimuli in all experiments. In all experiments stimuli were delivered for 500 ms and during stimulations the continuous flow was maintained at 2,000 ml/min. A continuous and humidified airstream (2,000 ml/min) was delivered to the fly head throughout the experiment via an 8 mm diameter glass tube positioned 10 mm away from the preparation. For putrescine stimulation, 1 ml of odor solution (0 mM (air stimulus), 1 mM, 10 mM, 100 mM diluted in pure water) was filled in the odor delivery cup and the collected airspace odor was injected into the main airstream for 500 ms without changing airstream strength. PID measurements suggested that 100 mM corresponded to ~10 ppm of odor and therefore compared best to the optimal concentration used in behavioral experiments. To measure the fluorescent intensity change, the ROI was delineated by hand and the resulting time trace was used for further analysis. To calculate the normalized change in the relative fluorescence intensity, we used the following formula: ΔF/F = 100(Fn − F0)/F0, where Fn is the nth frame after stimulation and F0 is the averaged basal fluorescence of 15 frames before stimulation. The peak fluorescence intensity change is calculated as the mean of normalized trace over a 2 s time window during the stimulation period. The pseudocolored images were generated in MATLAB using a custom written program. All analysis and statistical tests were done using Excel and GraphPad6 Prism softwares, respectively.

Imaging with taste stimuli was performed in a similar setup as described above with some modifications. The flies expressing GCaMP-fluorescence under IR76b-Gal4 were prepared according to a method previously described [75]. The proboscis of the fly was pulled out by suction and fixed by gluing to prevent it from going back into the head capsule. For taste stimulation, taste stimuli were diluted in distilled water and delivered by a custom-build syringe delivery system to the proboscis. Distilled water (control), 1 mM, 10 mM, and 100 mM putrescine were applied, respectively. Application of the stimulus was monitored by a stereomicroscope. A drop of taste was delivered to touch the labellum. The stimulus was applied for 1 s after the start of each measurement. Imaging of leg IR76b taste neurons was carried out as previously described [76]. Briefly, legs were detached from the body of the fly and glued to double-sided tape on a slide. 100 μl of water were added to the free lower segments of the leg to help focus the preparation on the IR76b neurons. For stimulation, different concentrations of 100 μl polyamine solution were added as 2 x to the water. All analysis and statistical tests were done using Excel and GraphPad6 Prism softwares as described above.

#### Electrophysiology

Tip recordings were carried out as previously described tip [77] with minor modifications. Female flies at 5–7 d after eclosion were used for all experiments. Legs and wings were removed to reduce movement. For recording, the flies were wedged into the narrow neck of a 200 μl pipette tip. The proboscis was extended and pasted by double-sided tape on a cover slide. The tip of a glass micropipette was used to hold the proboscis in a stable position. A reference electrode containing 0.01 mM KCL was inserted into the eye of the fly. The recording electrode consisted of a fine glass pipette (10–15 μm tip diameter) and a silver wire connected to an amplifier. The recording electrode played the dual function of recording and container for the stimulus. Recording started the moment that the recording electrode contacted the tip of the sensillum. The polyamine solution used for stimulation contained 30 mM tricholine citrate (TCC) as the electrolyte to suppress responses from the osmolarity-sensitive taste neuron. To avoid desensitization, stimuli were given at least 3 min apart. In all recordings, concentrations were increased sequentially and a control stimulus without polyamine was applied first in all cases. Recordings were performed on L-type and S-type sensilla on the labial palp. The recording electrode was connected to an amplifier Multiclamp 700B, and the AC signals (10–2,800 Hz) were recorded for 2–3 s, starting before stimulation, recorded and analyzed using Clampex10.3 (Digidata 1440A). The responses of neuron firing were calculated by counting the number of action potentials from 200 to 700 ms after initial contact, as previously reported [74].

The numerical data used in all main and supplementary figures are included in S1 Data.

## Supporting Information

S1 Data The excel spreadsheet contains, in separate sheets, the underlying numerical data and statistical analysis for the following figures with their relative panels: Fig 1, Fig 2, Fig 3, Fig 4, Fig 5, Fig 6, S1 Fig, S2 Fig, S3 Fig, S4 Fig, S5 Fig, S6 Fig, S7 Fig and S8 Fig. (XLSX)

S1 Fig Olfactory attraction to polyamines. (A) Dose-dependent (0.001–1,000 mM) olfactory preference of Canton S flies to putrescine and cadaverine. Box plots show median and upper/lower quartiles (*n* = 8, 60 ♀/trial, 30 ♀ and 30 ♂). (B) Analogous to group olfactory behavior, single flies chose polyamine side over non-polyamine side in T-maze assay. Graphs show preference of polyamine over non-polyamine side by single fly in 30 T-maze trials. (C) Polyamine-associated attraction of *Drosophila* is dependent on the main olfactory organ, the antenna. Bars show olfactory PI of wild type flies with or without antenna to 1 mM putrescine and cadaverine in the T-maze assay. Box plots show median and upper/lower quartiles (*n* = 8, 60 ♀/trial, 30 ♀ and 30 ♂). (D) ORs are not required for polyamine attraction. Bars show olfactory PI of *Orco-/-* flies to putrescine and cadaverine. Box plots show median and upper/lower quartiles (*n* = 8, 60 ♀/trial, 30 ♀ and 30 ♂). (E) IRs mediate olfactory attraction to polyamines. Bars show olfactory PI of control (*wt*: *eyflp; FRT82B/FRT82B cell lethal*) and mosaic atonal mutant (*ato-/-: eyflp; FRT82B ato[1]/FRT82B cell lethal*) flies to putrescine and cadaverine in the T-maze assay. Box plots show median and upper/lower quartiles (*n* = 8, 60 ♀/trial, 30 ♀ and 30 ♂). (F) Olfactory PI of putative candidate receptor mutants (*IR31a-/-*, *IR75a-/-*, *IR75d-/-*, *IR84a-/-* and *IR92a-/-*) for polyamine detection in the T-maze assay. Box plots show median and upper/lower quartiles (*n* = 8, 60 ♀/trial, 30 ♀ and 30 ♂). All *p*-values were calculated via standard *t* test (ns > 0.05, *p ≤ 0.05, **p ≤ 0.01, ***p ≤ 0.001).
(TIF)

S2 Fig IR76b-Gal4 and IR76b-QF show highly overlapping expression pattern. Comparison of GFP/RFP signals in *IR76b-QF;QUAS-mdTomato-3xHa* and *IR76b-Gal4;UASmCD8GFP* flies. Neurons in legs, antenna, labellum, and wings always show both GFP and RFP staining. The expression is by and large overlapping with few exceptions where green cells appear to be stained more strongly than red cells. Furthermore, the distribution of the fluorescence within the cells is different because of the nature of the respective reporter protein. Confocal images were taken at an Olympus Confocal microscope. Step size 0.5 μM. Single sections or small stacks are shown.
(TIF)

S3 Fig Non-Ir41a IR76b-expressing OSNs do not respond to putrescine. (A) Prestimulation fluorescence micrograph showing IR76b OSN axon-innervated glomeruli. VC5 is innervated by IR41a OSNs, which are polyamine responsive. The indicated ROI marks another IR76b innervated glomerulus that was analyzed for a putative response to putrescine. (B) Quantification of peak ΔF responses in mutant (*IR76b*^*1/1*^) and control flies that express *UAS-GCaMP6f* under the control of *IR76b-Gal4*. Boxes show median and upper/lower quartiles, and whiskers show minimum/maximum values. *p* > 0.05 by unpaired *t* test with Welch correction (*n* = 6). (C) Average activity trace of non-VC5 glomerulus. The gray bar represents the 0.5 second stimulation period. Dark colored line is the average response, and the light shade is the SEM.
(TIF)

S4 Fig IR76b receptor is required for oviposition preference. (A) Average total number of eggs after 16 h in oviposition assay of Canton S females. Female Canton S flies prefer to lay eggs on the control side (1% low melting agarose only, shown in gray) compared to polyamine side (shown in orange). The number of eggs is averaged for each stimulus (*n*v = v8 ± SEM, 60 ♀ flies/trial). (B) Dose-dependent (0.001–1,000 mM) oviposition preference of Canton S to putrescine and cadaverine (magenta: putrescine, green: cadaverine, gray: agarose). Box plots show median and upper/lower quartiles (*n* = 8, 60 ♀/trial). (C) Average number of eggs on stimulus and nonstimulus (1% low melting agarose, shown in gray bar) sites at different concentrations in oviposition assay. (D) Average total number of eggs in oviposition assay of single Canton S female fly (*n* = 30 ± SEM, 1 ♀ flies/trial). (E) Average total number of eggs in oviposition assay of Canton S female fly (*n* = 8 ± SEM, 60 ♀ flies/trial). (F) Addition of polyamines significantly increases the attractiveness of sugar as egg-laying substrate compared to sugar alone. Graphs show number of eggs in the presence and absence of polyamines (putrescine) after 16 h, number of eggs are averaged for each stimulus (*n* = 8 ± SEM, 10 ♀ flies/trial). (G) Polyamine-triggered oviposition choice behavior depends on the sense of taste (labellum). Bars show average total number of eggs for antenna, legs, wings, and labellum ablated flies (− depicts ablation, + shows non ablation). (*n* = 8 ± SEM, 60 ♀ flies/trial). (H) Egg numbers of 16 h oviposition assay of *Poxn* mutants (Poxn^-/-^) and Poxn ^-/-^ rescues SuperA. (I) Oviposition PI of odorant coreceptor mutant (*Orco*^*-/-*)^ and mutants of putative ionotropic coreceptor mutants (*IR8a*^*-/-*^, *IR25a*^*-/-*^, and *IR76b*^*-/-*)^ to polyamines. The gray box serves as a control and shows that females show no side preference on plain agar plates. Box plots show median and upper/lower quartiles (*n* = 8, 60 ♀/trial). (J) Egg numbers of S4I Fig. PIs are averaged (*n* = 8 ± SEM, 60 ♀ flies/trial). (K) Egg numbers of 16 h oviposition assay of different mutants and controls corresponding to Fig 3H–3I. Number of eggs are averaged (*n* = 8 ± SEM). (L) Average number of eggs after 16 h oviposition assay corresponding to Fig 3J. Number of eggs is averaged (*n* = 8 ± SEM).
(TIF)

S5 Fig IR76b and Gr66a are not coexpressed in labellar taste neurons. Expression analysis of IR76b (*IR76b-QF;QUAS-mdTomato-3xHa*) and Gr66a (*Gr66a-Gal4;UASmCD8GFP*) in proboscis, brain (SEZ), and legs. No coexpression could be observed in neurons of the labellum and axons projecting from these to the SEZ innervated neighboring regions and did not overlap. Coexpressing cells were occasionally found in the leg. The legs, however, were redundant for fly’s taste preference behavior. Confocal images were taken at an Olympus Confocal microscope. Step size 0.5 μM. Single sections or small stacks are shown.
(TIF)

S6 Fig IR76b neurons mediate responses to low salt concentration. (A) Quantification of peak responses of GCaMP6f-fluorescence (in %ΔF/F) in the ROI 1 and ROI 2 areas, respectively, when *IR76b-Gal4;UAS-GCaMP6f* female flies were stimulated with distilled water or 50 mM NaCl_2_ (*n* = 6 ± SEM). (B) Representative image of calcium responses of a leg expressing GCaMP under the control of IR76b-Gal4 stimulated with polyamine. (C) Average response trace of tarsal IR76b neurons (*n* = 8 ± SEM). (D) Tarsal IR76b neurons respond to high concentrations of polyamines (*n* = 8 ± SEM). (E) Quantification of peak responses (in %ΔF/F) of *IR76b* mutant and heterozygous controls in the ROI 1 and ROI 2 areas, respectively to 50 mM NaCl_2_ stimulation (*n* = 6 ± SEM). Boxes show median and upper/lower quartiles, and whiskers show minimum/maximum values. All *p*-values were calculated via Student’s *t* test (ns > 0.05, *p ≤ 0.05, **p ≤ 0.01, ***p ≤ 0.001).
(TIF)

S7 Fig Egg numbers after 3 h of egg laying. (A–D) Graphs display the number of eggs females laid in the first 3 h of a position–oviposition assay on control (1% low melting agarose) or stimulus (agarose plus polyamines) site corresponding to Fig 5. Note that the low number of eggs in some test lines reflects the slow start of oviposition due to genetic or other manipulations. Egg numbers caught up later significantly (see for instance S4 Fig). However, it is important to interpret some of the oviposition preferences with caution due to the low number of eggs. Number of eggs are averaged for each time point (*n* = 8 ± SEM, 60 ♀ flies/trial).
(TIF)

S8 Fig Structural similarity of NMDA receptor subunit NR1 and polyamine receptors. (A) Schematic presentation of putative structure of the NR1 subunit of the NMDA receptor. Acidic amino acids have been marked in red. In particular, several acidic residues in domains S1, S2, R1, and R2 have been implicated in polyamine-mediated potentiation [69–71]. (B) Putative structure of IR76b. (C) Putative structure of IR41a. (D) Structure comparison of NR1 with IR76b, (E) with IR41a, and (F) structure comparison between IR41a and IR76b.
(TIF)

## Abbreviations

AL: antennal lobe; *ato*, *atonal*
GR: gustatory receptor
GRN: gustatory receptor neuron
iGluRs: ionotropic glutamate receptors
IR: ionotropic receptor
MFC: mass flow controller
NMDA: N-methyl-D-aspartate receptor
OR: olfactory receptor
Orco: OR coreceptor
OSN: olfactory sensory neuron
PI: preference index
PID: photoionization detector
PN: projection neuron
ROI: region of interest
SEM: standard error of the mean
SEZ: subesophageal zone
SSR: single sensillum recording
TAAR13c: trace amine-associated receptor 13c
TCC: tricholine citrate
