# Correction: Ionotropic Chemosensory Receptors Mediate the Taste and Smell of Polyamines

**DOI:** 10.1371/journal.pbio.1002505

**Published:** 2016-06-21

**Authors:** The PLOS Biology Staff

## Notice of Republication

This article was republished on May 19^th^, 2016 to correct poor figure quality in the PDF version, which was introduced during the typesetting process. The publisher apologizes for these issues with the figure quality. Please download this article again to view the updated version.

## References

[1] Hussain A, Zhang M, Üçpunar HK, Svensson T, Quillery E, Gompel N, et al (2016) Ionotropic Chemosensory Receptors Mediate the Taste and Smell of Polyamines. PLoS Biol 14(5): e1002454 doi:10.1371/journal.pbio.1002454 27145030 10.1371/journal.pbio.1002454 PMC4856413

