# Correction: Ionotropic Chemosensory Receptors Mediate the Taste and Smell of Polyamines

**DOI:** 10.1371/journal.pbio.1002624

**Published:** 2018-03-14

**Authors:** Ashiq Hussain, Mo Zhang, Habibe K. Üçpunar, Thomas Svensson, Elsa Quillery, Nicolas Gompel, Rickard Ignell, Ilona C. Grunwald Kadow

The following information is missing from the Funding section: This study was supported by an ERC starting grant to ICGK (FlyContext, 637472, https://erc.europa.eu).
