# Supplementary figures and images for: Ionotropic Chemosensory Receptors Mediate the Taste and Smell of Polyamines
Source: PLoS Biol. 2016 May 4;14(5):e1002454. doi: 10.1371/journal.pbio.1002454 (PMC4856413; doi:10.1371/journal.pbio.1002454)

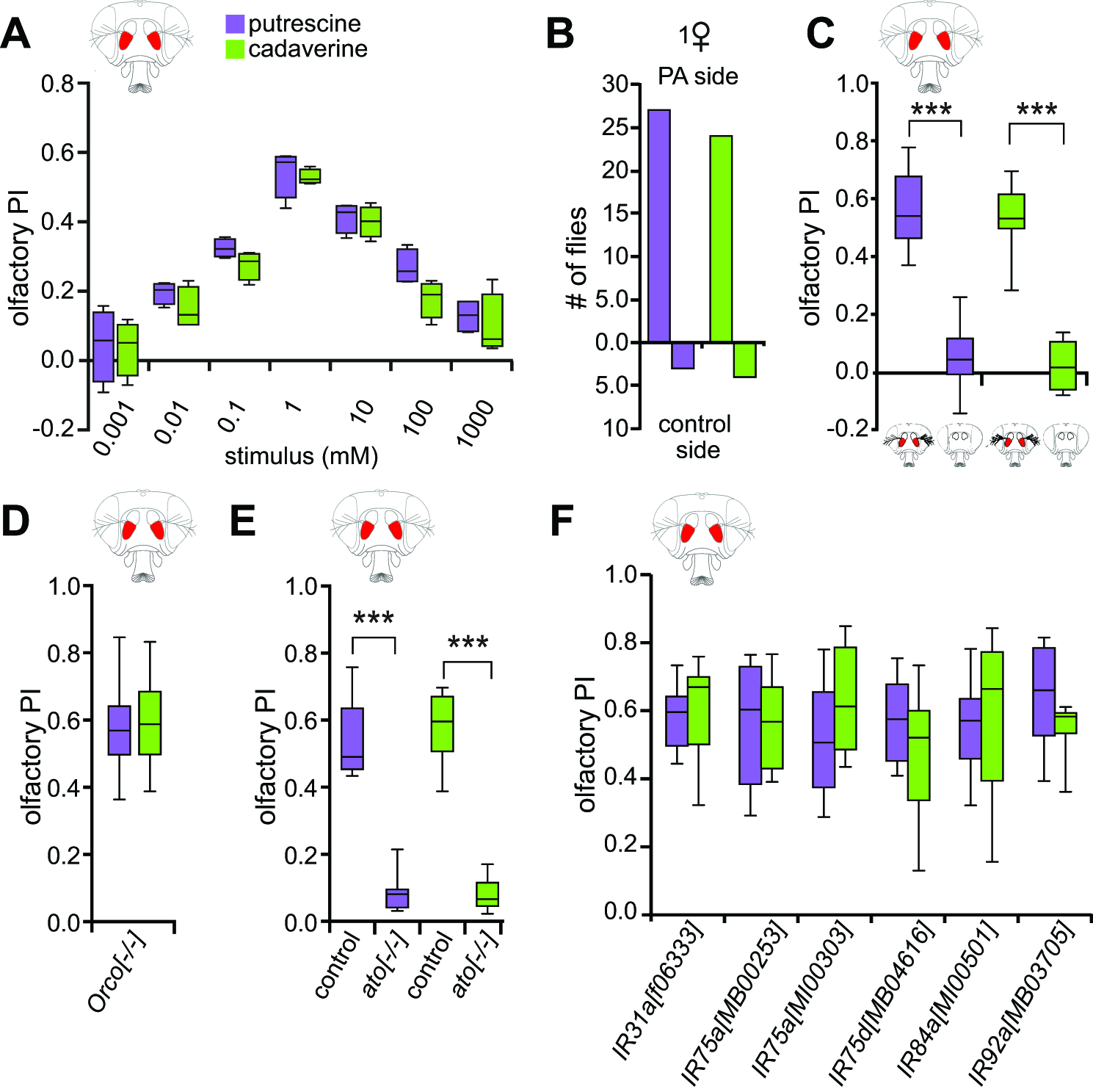

Supplement: S1 Fig — (A) Dose-dependent (0.001–1,000 mM) olfactory preference of Canton S flies to putrescine and cadaverine. Box plots show median and upper/lower quartiles (n = 8, 60 ♀/trial, 30 ♀ and 30 ♂). (B) Analogous to group olfactory behavior, single flies chose polyamine side over non-polyamine side in T-maze assay. Graphs show preference of polyamine over non-polyamine side by single fly in 30 T-maze trials. (C) Polyamine-associated attraction of Drosophila is dependent on the main olfactory organ, the antenna. Bars show olfactory PI of wild type flies with or without antenna to 1 mM putrescine and cadaverine in the T-maze assay. Box plots show median and upper/lower quartiles (n = 8, 60 ♀/trial, 30 ♀ and 30 ♂). (D) ORs are not required for polyamine attraction. Bars show olfactory PI of Orco-/- flies to putrescine and cadaverine. Box plots show median and upper/lower quartiles (n = 8, 60 ♀/trial, 30 ♀ and 30 ♂). (E) IRs mediate olfactory attraction to polyamines. Bars show olfactory PI of control (wt: eyflp; FRT82B/FRT82B cell lethal) and mosaic atonal mutant (ato-/-: eyflp; FRT82B ato[1]/FRT82B cell lethal) flies to putrescine and cadaverine in the T-maze assay. Box plots show median and upper/lower quartiles (n = 8, 60 ♀/trial, 30 ♀ and 30 ♂). (F) Olfactory PI of putative candidate receptor mutants (IR31a-/-, IR75a-/-, IR75d-/-, IR84a-/- and IR92a-/-) for polyamine detection in the T-maze assay. Box plots show median and upper/lower quartiles (n = 8, 60 ♀/trial, 30 ♀ and 30 ♂). All p-values were calculated via standard t test (ns > 0.05, *p ≤ 0.05, **p ≤ 0.01, ***p ≤ 0.001). (TIF) [file pbio.1002454.s002.tif]

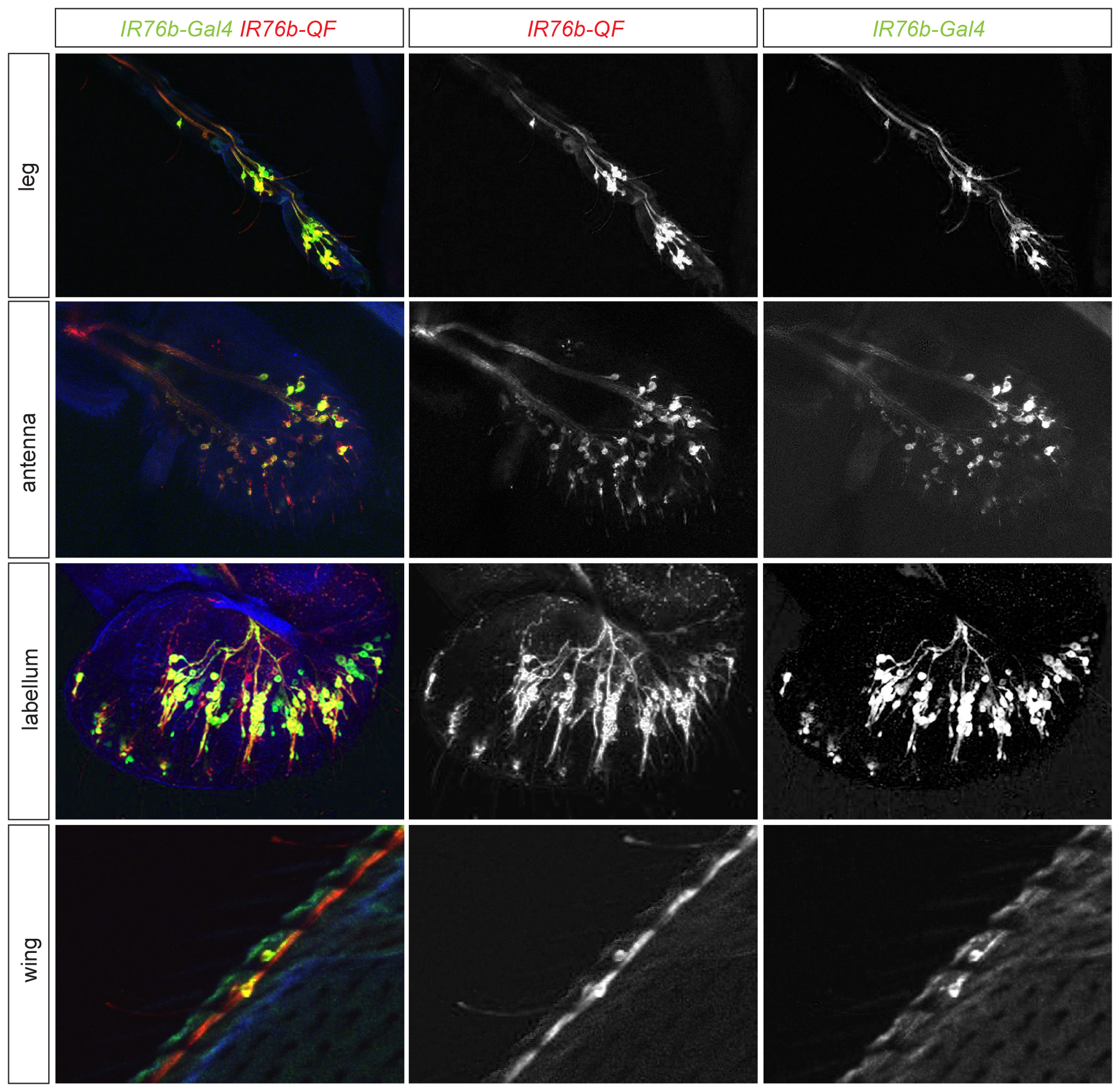

Supplement: S2 Fig — Comparison of GFP/RFP signals in IR76b-QF;QUAS-mdTomato-3xHa and IR76b-Gal4;UASmCD8GFP flies. Neurons in legs, antenna, labellum, and wings always show both GFP and RFP staining. The expression is by and large overlapping with few exceptions where green cells appear to be stained more strongly than red cells. Furthermore, the distribution of the fluorescence within the cells is different because of the nature of the respective reporter protein. Confocal images were taken at an Olympus Confocal microscope. Step size 0.5 μM. Single sections or small stacks are shown. (TIF) [file pbio.1002454.s003.tif]

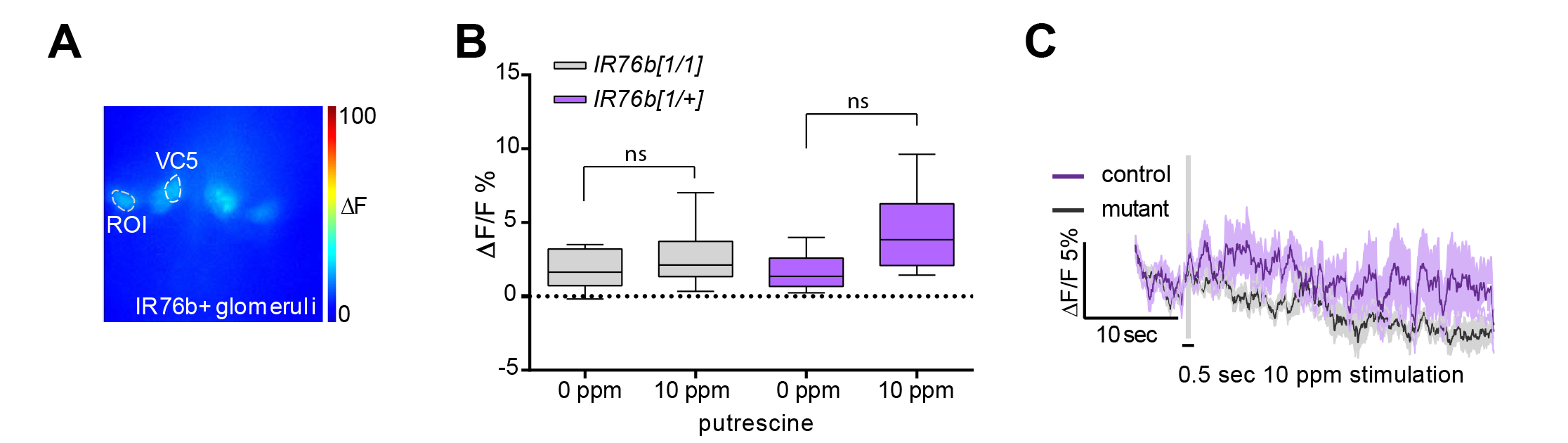

Supplement: S3 Fig — (A) Prestimulation fluorescence micrograph showing IR76b OSN axon-innervated glomeruli. VC5 is innervated by IR41a OSNs, which are polyamine responsive. The indicated ROI marks another IR76b innervated glomerulus that was analyzed for a putative response to putrescine. (B) Quantification of peak ΔF responses in mutant (IR76b1/1) and control flies that express UAS-GCaMP6f under the control of IR76b-Gal4. Boxes show median and upper/lower quartiles, and whiskers show minimum/maximum values. p > 0.05 by unpaired t test with Welch correction (n = 6). (C) Average activity trace of non-VC5 glomerulus. The gray bar represents the 0.5 second stimulation period. Dark colored line is the average response, and the light shade is the SEM. (TIF) [file pbio.1002454.s004.tif]

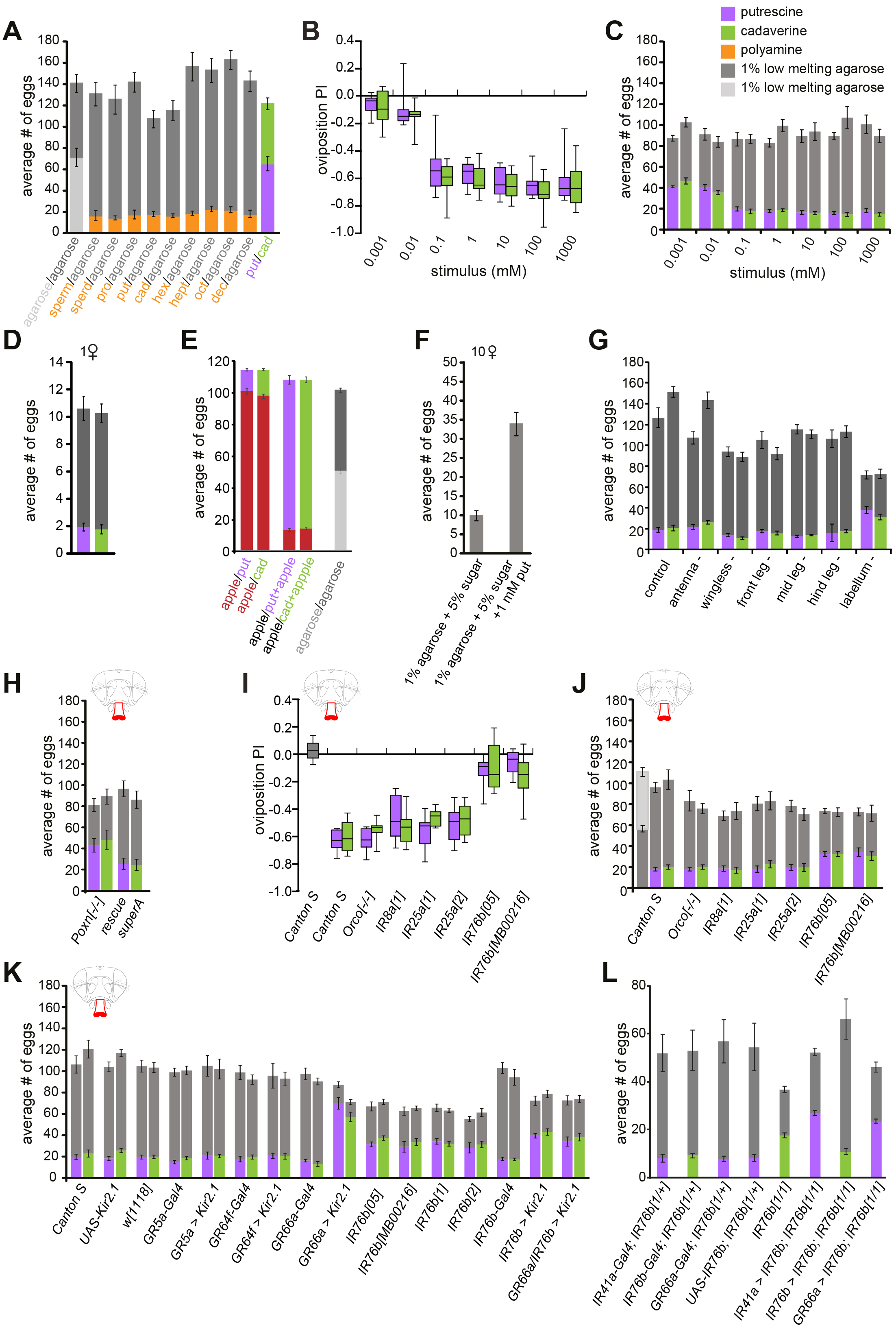

Supplement: S4 Fig — (A) Average total number of eggs after 16 h in oviposition assay of Canton S females. Female Canton S flies prefer to lay eggs on the control side (1% low melting agarose only, shown in gray) compared to polyamine side (shown in orange). The number of eggs is averaged for each stimulus (nv = v8 ± SEM, 60 ♀ flies/trial). (B) Dose-dependent (0.001–1,000 mM) oviposition preference of Canton S to putrescine and cadaverine (magenta: putrescine, green: cadaverine, gray: agarose). Box plots show median and upper/lower quartiles (n = 8, 60 ♀/trial). (C) Average number of eggs on stimulus and nonstimulus (1% low melting agarose, shown in gray bar) sites at different concentrations in oviposition assay. (D) Average total number of eggs in oviposition assay of single Canton S female fly (n = 30 ± SEM, 1 ♀ flies/trial). (E) Average total number of eggs in oviposition assay of Canton S female fly (n = 8 ± SEM, 60 ♀ flies/trial). (F) Addition of polyamines significantly increases the attractiveness of sugar as egg-laying substrate compared to sugar alone. Graphs show number of eggs in the presence and absence of polyamines (putrescine) after 16 h, number of eggs are averaged for each stimulus (n = 8 ± SEM, 10 ♀ flies/trial). (G) Polyamine-triggered oviposition choice behavior depends on the sense of taste (labellum). Bars show average total number of eggs for antenna, legs, wings, and labellum ablated flies (− depicts ablation, + shows non ablation). (n = 8 ± SEM, 60 ♀ flies/trial). (H) Egg numbers of 16 h oviposition assay of Poxn mutants (Poxn-/-) and Poxn -/- rescues SuperA. (I) Oviposition PI of odorant coreceptor mutant (Orco-/-) and mutants of putative ionotropic coreceptor mutants (IR8a-/-, IR25a-/-, and IR76b-/-) to polyamines. The gray box serves as a control and shows that females show no side preference on plain agar plates. Box plots show median and upper/lower quartiles (n = 8, 60 ♀/trial). (J) Egg numbers of S4I Fig. PIs are averaged (n = 8 ± SEM, 60 ♀ flies/trial). [file pbio.1002454.s005.tif]

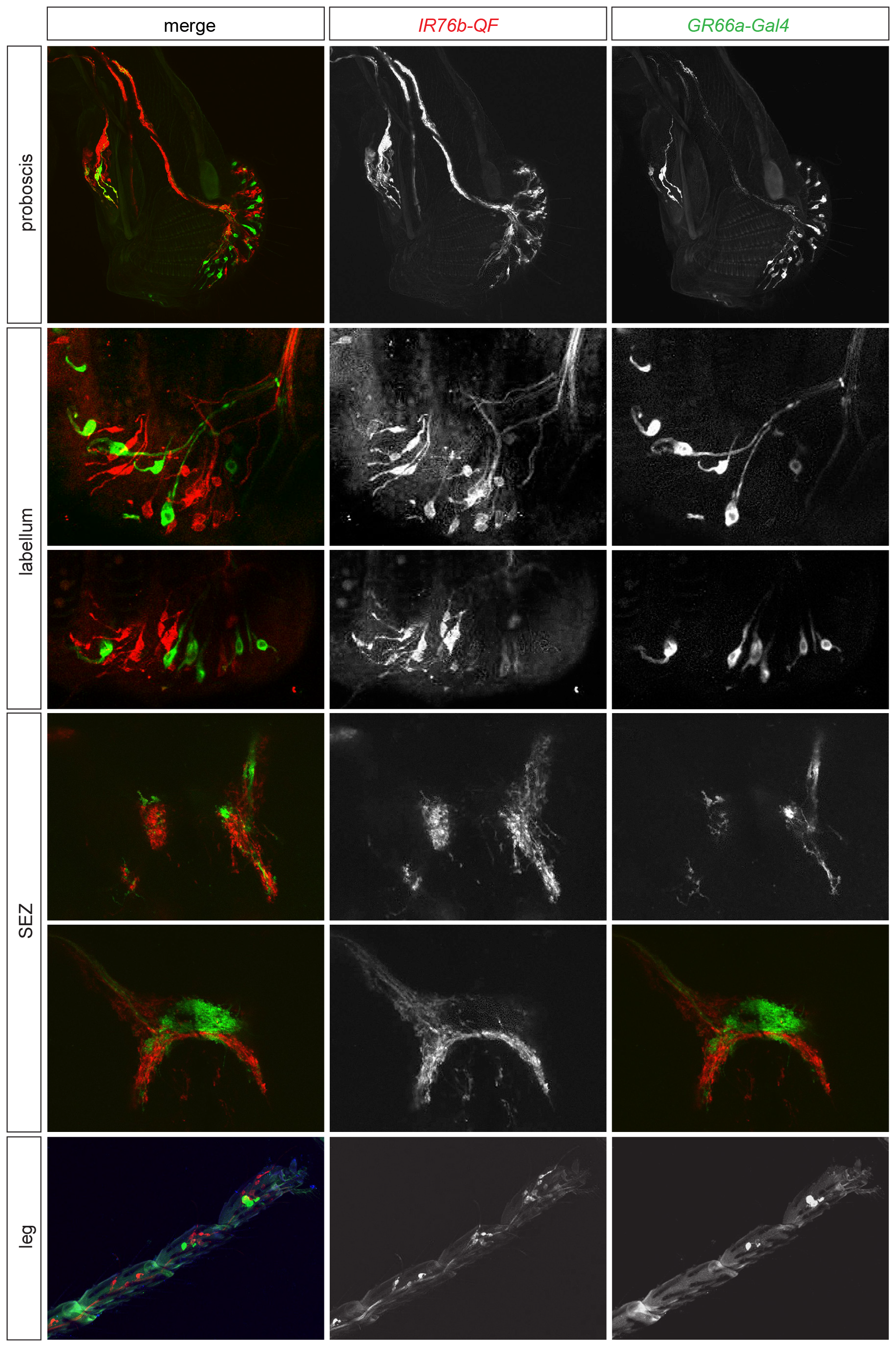

Supplement: S5 Fig — Expression analysis of IR76b (IR76b-QF;QUAS-mdTomato-3xHa) and Gr66a (Gr66a-Gal4;UASmCD8GFP) in proboscis, brain (SEZ), and legs. No coexpression could be observed in neurons of the labellum and axons projecting from these to the SEZ innervated neighboring regions and did not overlap. Coexpressing cells were occasionally found in the leg. The legs, however, were redundant for fly’s taste preference behavior. Confocal images were taken at an Olympus Confocal microscope. Step size 0.5 μM. Single sections or small stacks are shown. (TIF) [file pbio.1002454.s006.tif]

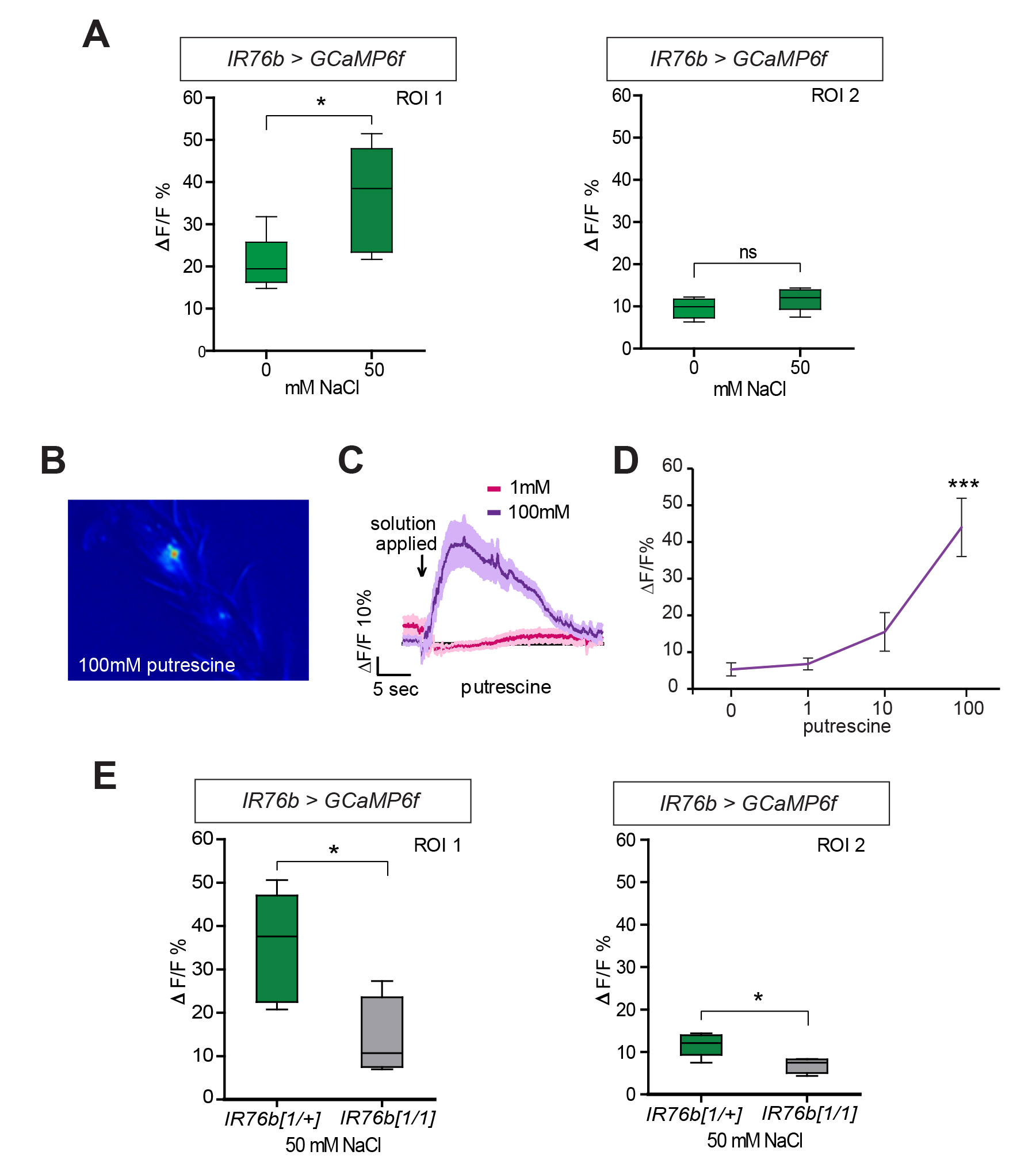

Supplement: S6 Fig — (A) Quantification of peak responses of GCaMP6f-fluorescence (in %ΔF/F) in the ROI 1 and ROI 2 areas, respectively, when IR76b-Gal4;UAS-GCaMP6f female flies were stimulated with distilled water or 50 mM NaCl2 (n = 6 ± SEM). (B) Representative image of calcium responses of a leg expressing GCaMP under the control of IR76b-Gal4 stimulated with polyamine. (C) Average response trace of tarsal IR76b neurons (n = 8 ± SEM). (D) Tarsal IR76b neurons respond to high concentrations of polyamines (n = 8 ± SEM). (E) Quantification of peak responses (in %ΔF/F) of IR76b mutant and heterozygous controls in the ROI 1 and ROI 2 areas, respectively to 50 mM NaCl2 stimulation (n = 6 ± SEM). Boxes show median and upper/lower quartiles, and whiskers show minimum/maximum values. All p-values were calculated via Student’s t test (ns > 0.05, *p ≤ 0.05, **p ≤ 0.01, ***p ≤ 0.001). (TIF) [file pbio.1002454.s007.tif]

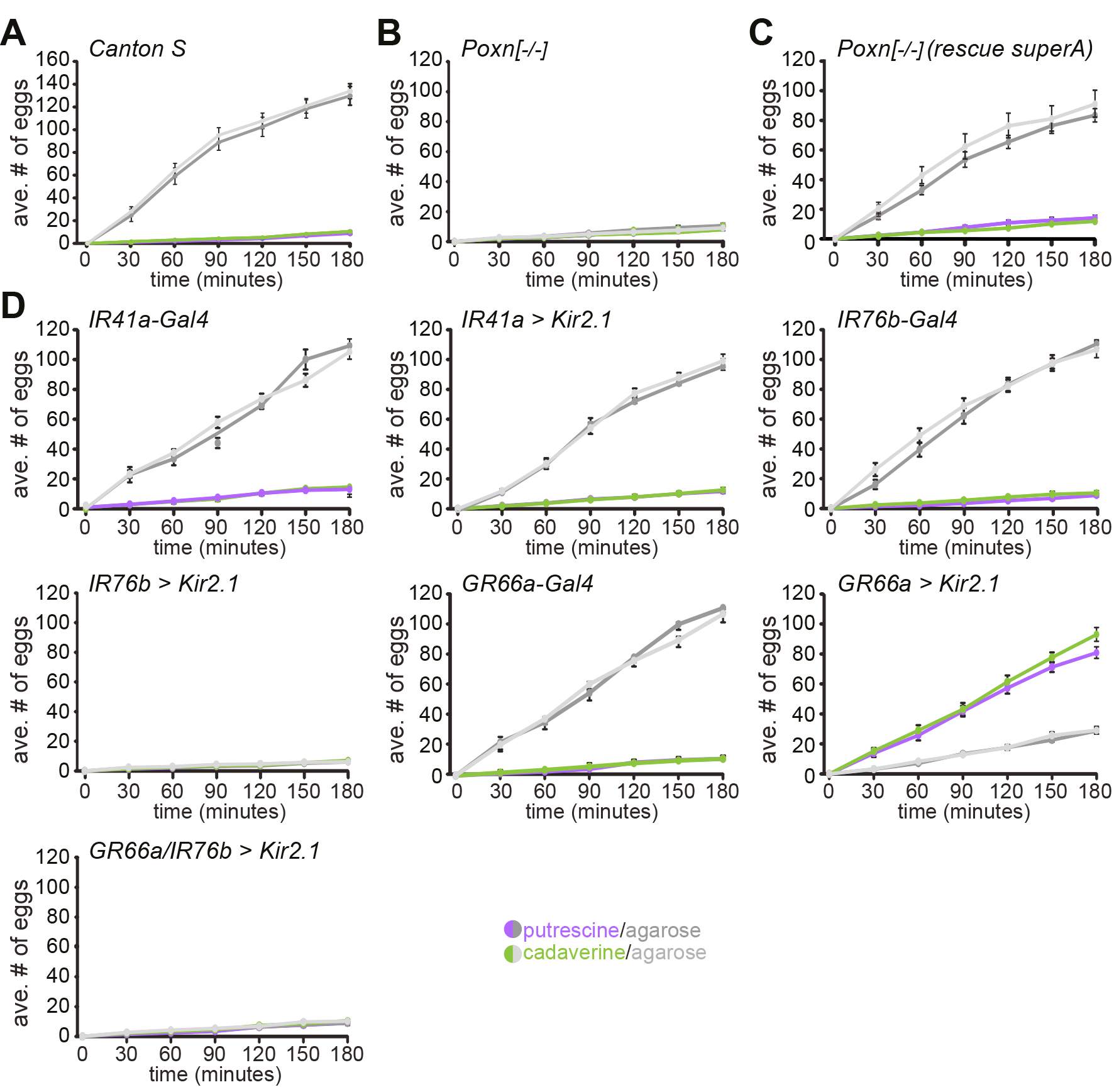

Supplement: S7 Fig — (A–D) Graphs display the number of eggs females laid in the first 3 h of a position–oviposition assay on control (1% low melting agarose) or stimulus (agarose plus polyamines) site corresponding to Fig 5. Note that the low number of eggs in some test lines reflects the slow start of oviposition due to genetic or other manipulations. Egg numbers caught up later significantly (see for instance S4 Fig). However, it is important to interpret some of the oviposition preferences with caution due to the low number of eggs. Number of eggs are averaged for each time point (n = 8 ± SEM, 60 ♀ flies/trial). (TIF) [file pbio.1002454.s008.tif]

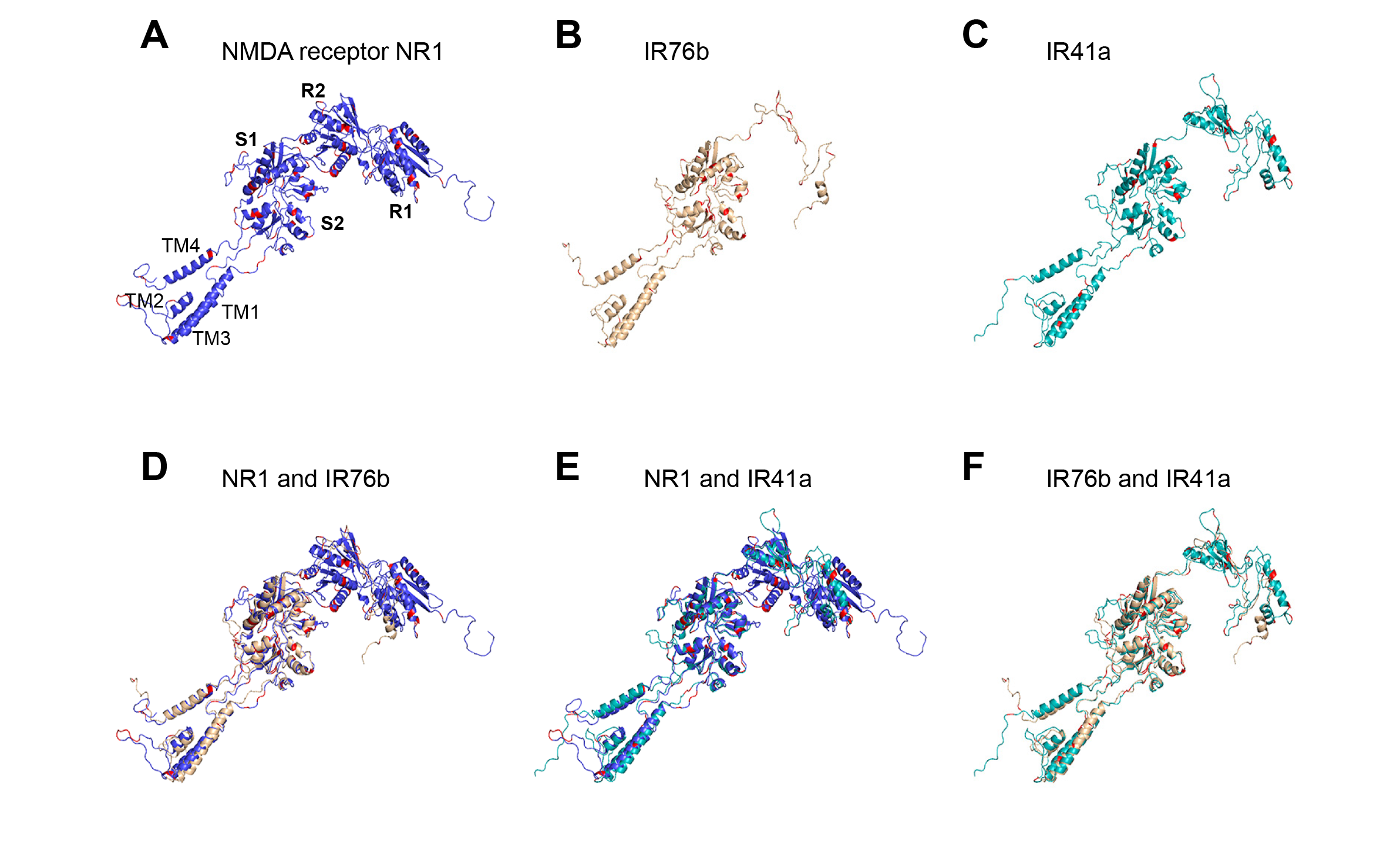

Supplement: S8 Fig — (A) Schematic presentation of putative structure of the NR1 subunit of the NMDA receptor. Acidic amino acids have been marked in red. In particular, several acidic residues in domains S1, S2, R1, and R2 have been implicated in polyamine-mediated potentiation [69–71]. (B) Putative structure of IR76b. (C) Putative structure of IR41a. (D) Structure comparison of NR1 with IR76b, (E) with IR41a, and (F) structure comparison between IR41a and IR76b. (TIF) [file pbio.1002454.s009.tif]
